# Genomic insights into the taxonomy, safety and biotechnological potential of Aphanizomenon flos-aquae and related Nostocales

**DOI:** 10.1099/mgen.0.001832

**Published:** 2026-09-23

**Authors:** Gabriel D. Scoglio, David H. Green, Harry O. Jackson, Saul Purton

**Affiliations:** 1 Department of Structural and Molecular Biology, University College London, Gower Street, London WC1E 6BT, UK; 2 Culture Collection of Algae and Protozoa, Scottish Association for Marine Science, Oban, Argyll, PA37 1QA, UK

**Keywords:** *Aphanizomenon flos-aquae* (*AFA*), *btu* operon, comparative genomics, cyanotoxin, Klamath AFA, natural competence, Nostocales, vitamin B12

## Abstract

The *Nostocales* are filamentous cyanobacteria that include bloom-forming, nitrogen-fixing species with major ecological and biotechnological relevance. Owing to their metabolic diversity, nitrogen-fixing capabilities and nutritional profile, they hold potential for various biotechnological applications including the production of nutraceuticals and biofertilizers. However, the capacity of certain *Nostocales* species to produce cyanotoxins also raises health concerns. This study provides a comparative bioinformatic analysis of 161 *Nostocales* genomes from the National Center for Biotechnology Information (NCBI) and three newly sequenced *Aphanizomenon flos-aquae* (*AFA*) strains, including Klamath *AFA*, with a focus on taxonomy, cyanotoxin-associated biosynthetic gene clusters (BGCs), natural competence, restriction-modification systems and vitamin B_12_ metabolism. Our analysis supports reassessment of several *AFA*-labelled genomes and refines the boundaries of the core *AFA* cluster. Cyanotoxin-associated BGCs, specifically for anatoxin-a, saxitoxin, cylindrospermopsin and microcystin/nodularin, were detected in only 7.3% of the genomes. Natural competence genes required for horizontal gene transfer were found in 89.6% of the genomes, although the presence of multiple restriction-modification system-associated proteins in *Nostocales* species, especially *Aphanizomenon, Anabaena, Dolichospermum* and *Nostoc* sp. may limit foreign gene acquisition. Additionally, ~30% of the genomes harbour a shared *btu* operon arrangement associated with B_12_ import, observed here across multiple *Nostocales* genera. B_12_ content in the *AFA* strains was measured at ~0.4–0.5 µg g^−1^ dry biomass and is likely linked to associated B_12_-producing bacteria. Fluorescent B_12_ analogue uptake experiments in NIES 4052 and Klamath *AFA* support a model for the uptake of exogenous B_12_ in these strains. Together with the detection of cobalamin-producing commensal bacteria, this suggests that B_12_ in *AFA* biomass may be linked to cyanobacterium–bacteria interactions. Overall, this study provides a genomic and experimental framework for evaluating the taxonomy, safety, genetic tractability and nutritional potential of *AFA* and related *Nostocales*.

**Impact Statement**

Through a combined analysis of 161 publicly available genomes and three newly sequenced *Aphanizomenon flos-aquae* (*AFA*) strains, this study delivers one of the most comprehensive genomic evaluations of *AFA* and related *Nostocales* strains to date. By refining the placement of *AFA*-labelled genomes, defining a conserved cyanobacterial *btu* operon and mapping the distribution of putative cyanotoxin-associated biosynthetic clusters, the work provides a clearer foundation for strain identification and safety assessment. The finding that natural competence genes are widespread, yet are accompanied by frequent restriction-modification system-associated proteins, adds mechanistic context to the limited genetic tractability of these strains. Functional B_12_ uptake experiments further support the genomic findings and help explain the presence of B_12_ in non-axenic *AFA* biomass. Together, these results connect taxonomy, genomics and phenotype in a unified framework, strengthening our understanding of the biotechnological potential of *AFA* and related *Nostocales* strains in sustainable nutrition and cyanobacterial biotechnology.

## Data Summary

Genome assemblies for the three *Aphanizomenon flos-aquae* (*AFA*) strains analysed in this study, CCAP 1401/3, NIES 4052 and Klamath *AFA*, are publicly available in the NCBI Assembly database under BioProject PRJNA1207407. The corresponding assembly accessions are GCA_060050125.1 for Klamath *AFA*, GCA_060050165.1 for NIES 4052 *AFA*, and GCA_060050145.1 for CCAP 1401/3 *AFA*. Individual BioSample and SRA accession numbers are provided in Table S1 (available in the online Supplementary Material). Annotated genome assemblies are openly available through Zenodo at https://doi.org/10.5281/zenodo.20529214. All additional genomic datasets analysed in this study, including the 161 publicly available *Nostocales* genomes, are referenced with accession IDs in Table S2a. All bioinformatics data and scripts used within this study are provided within the article or its supplementary files. Any further supporting datasets or intermediate files are available from the corresponding author upon reasonable request.

## Introduction

Cyanobacteria are ancient photosynthetic prokaryotes integral to Earth’s nitrogen and oxygen cycles, with global distribution and remarkable ecological and biotechnological significance [1]. One notable cyanobacterial order is the *Nostocales*, which includes genera such as *Nostoc*, *Anabaena*, *Calothrix* and *Aphanizomenon,* under traditional cyanobacterial taxonomy [2]. These filamentous organisms are characterized by their ability to fix atmospheric nitrogen through specialized cells called heterocysts [3]. Additionally, many *Nostocales* species can form dormant cells termed akinetes that allow them to withstand extreme environmental conditions and re-establish populations when favourable conditions return [4]. These adaptive traits enable *Nostocales* strains to dominate nitrogen-poor freshwater systems, often resulting in dense blooms that impact ecosystems [2].

Natural *Nostocales* blooms have been exploited as valuable biomass for commercial applications, including as nutraceuticals, biofertilizers and aquaculture feed [5]. A notable example is the harvesting of *Aphanizomenon flos-aquae* (*AFA*) from Upper Klamath Lake (Oregon, USA), which produces up to 1,000 tonnes of biomass every year that is sold worldwide as a nutritional supplement [6]. *AFA* products and extracts (e.g. C-phycocyanin and β-phenylethylamine) have been reported to exhibit anti-inflammatory and antioxidant properties and to provide nutritional value [7–9]. Furthermore, toxicological investigations of Klamath *AFA* have consistently shown no evidence of cyanotoxin production [10, 11]. Despite this, the presence of cyanotoxin-producing *Nostocales* such as *Cuspidothrix issatschenkoi* or *Aphanizomenon gracile* raises significant health concerns [2]. These cyanotoxins include microcystins (MYCs), nodularins (NODs), anatoxins (ATXs), saxitoxins (SXTs) and cylindrospermopsins (CYRs) that have been linked to harmful algal blooms (HABs) and livestock poisonings [12–14].

Accurate taxonomy is therefore central to the safe commercial and biotechnological use of *AFA* and related *Nostocales* species. Misidentification of cyanotoxin-producing species has historically complicated the interpretation of *Nostocales* safety, particularly where toxin-producers such as *Aphanizomenon gracile* have been conflated with non-toxic *AFA* [2, 15]. Taxonomic ambiguity among *Nostocales* type species has hindered precise identification of toxin producers, resulting in potential over-regulation and missed biotechnological opportunities [16, 17]. Within this context, several recent phylogenomic studies have highlighted broader inconsistencies among *Nostocales* genera, particularly the close clustering of *Aphanizomenon*, *Dolichospermum* and *Anabaena* in whole-genome phylogenies [2, 18, 19]. These three genera have been collectively referred to as the ‘*ADA* clade’, and some authors have proposed unifying them into a single genus [12, 19, 20]. Nevertheless, clear morphological distinctions exist: *Anabaena* and *Dolichospermum* strains typically exhibit oval or barrel-shaped vegetative cells and heterocysts, whereas *AFA* possesses cylindrical cells and forms characteristic eye-visible bundled filamentous aggregates, known as fascicles that are absent in the other groups [2, 21]). This indicates that morphological and phylogenomic classifications do not fully coincide. These inconsistencies highlight the need to evaluate *AFA*-labelled strains within a comparative genomic framework, particularly when taxonomic identity is linked to safety assessment and biotechnological use.

Beyond taxonomy and safety, *Nostocales* have promising applications in sustainable biotechnology. Their nitrogen-fixing capabilities make them potential biofertilizer resources for enhanced agricultural productivity [22]. In parallel, genetic engineering could further expand their application by improving traits relevant to biomass productivity, nutrient delivery and the production of high-value compounds [23, 24]. Many *Nostocales* possess genes associated with natural competence for exogenous DNA uptake, allowing a potential genetic modification strategy for enhanced production of high-value compounds such as antimicrobials, pharmaceuticals and nutraceuticals [25–27]. The ability to genetically manipulate *AFA* is of particular interest because *AFA* biomass is already used for human consumption, although market approval of genetically modified cyanobacteria remains complex [28, 29]. However, the efficiency of transgene delivery into cyanobacterial genomes can be limited by restriction–modification system-associated proteins (R–M system-associated proteins) that degrade incoming DNA, making it important to assess natural competence genes alongside putative R–M system-associated proteins [30, 31].

A further unresolved feature of *AFA* biotechnology concerns vitamin B_12_. Cyanobacteria primarily produce pseudo-B_12_, an analogue with low binding affinity for human intrinsic factor and limited relevance for human absorption [32]. Klamath *AFA* biomass has been reported to contain a small but nutritionally relevant proportion of true B_12_, but this is thought to derive from co-habiting B_12_-producing commensal bacteria rather than from *AFA* [9, 33]. This is further supported by evidence that cyanobacteria possess B_12_ import systems encoded by *btu* genes and will preferentially use true B_12_ over pseudo-B_12_ as a cofactor [32, 34, 35].

The overarching aim of this study was to establish an integrated genomic and experimental framework for evaluating the biotechnological potential of *AFA* and related *Nostocales*. Specifically, we aimed to: (i) assess the genomic coherence and taxonomic placement of *AFA*-labelled genomes using genome statistics, phylogenomics, genome taxonomy database toolkit (GTDB-Tk) classification and average nucleotide identity (ANI) analysis; (ii) evaluate the distribution of putative cyanotoxin-associated biosynthetic gene clusters (BGCs), natural competence genes and R–M system-associated proteins across the 164 genomes; and (iii) investigate vitamin B_12_ metabolism in *AFA* by combining *btu* gene analysis, commensal bacterial pathway prediction, approximate B_12_/pseudo-B_12_ content quantification and fluorescent B_12_ analogue uptake experiments. Together, these analyses establish a comprehensive genomic and biotechnological framework for *AFA* and related *Nostocales* species, providing the taxonomic, safety-related and functional data needed to support their responsible use across a range of applications.

## Methods

### *AFA* strains, isolation and maintenance

*AFA* CCAP 1401/3 was obtained from the CCAP culture collection in Oban, Scotland (https://www.ccap.ac.uk/). The strain was originally isolated from Queen Elizabeth Reservoir, London, England, UK in 1970. *AFA* NIES 4052 was obtained from the NIES culture collection in Tsukuba, Japan (https://mcc.nies.go.jp/). It was originally isolated from Ponnuma Pond, Hamatonbetsu, Hokkaido, Japan in 2014. Klamath *AFA* was collected in 2022 by G.D.S. from Upper Klamath Lake, Oregon, USA. All *AFA* strains were maintained as unialgal non-axenic cultures in liquid Jaworski medium (JM; [36]) using a 16 h:8 h light/dark (L/D) cycle at 50 µmol photons m^−2^ s^−1^ at 25 °C and mixing from 80 to 120 r.p.m.

### Genomic DNA extraction and whole-genome sequencing

The *AFA* strains CCAP 1401/3*,* NIES 4052 and Klamath *AFA* were depleted of bacterial contaminants by three repeated steps of washing, filtration through 50 µm pore size membrane and low-speed centrifugation (2,800 ***g***) prior to DNA extraction. Genomic DNA was extracted from the strains using the GenElute™ Bacterial Genomic DNA Kits (Sigma-Aldrich), analysed by agarose gel electrophoresis and quantified using a NanoDrop ND-1000 Spectrophotometer (Thermo Scientific UK Ltd). Genomic regions encoding 16S rRNA and phycocyanin were amplified by PCR using primers specific to cyanobacteria and Q5 DNA polymerase (NEB). The 16S rRNA primers were 27F (AGAGTTTGATCCTGGCTCAG) and 809R (GCTTCGGCACGGCTCGGGTCGATA), whereas the phycocyanin primers were PCbF (GGCTGCTTGTTTACGCGACA) and PCaR (CCAGTACCACCAGCAACTAA) [37]. PCR products were purified from the reaction mix using the GeneJET PCR Purification Kit (Thermo Fisher) and sent for Sanger sequencing (Source Biosciences). DNA sequence identity was confirmed by blastn analysis against the NCBI nr database. Genomic DNA of all three *AFA* strains were submitted for whole-genome sequencing (WGS) using Illumina PE150 sequencing on NovaSeq 6000 (Novogene UK Ltd). Further to this, DNA from CCAP 1401/3 and Klamath *AFA* were sent to CD Genomics USA for sequencing by Oxford Nanopore Technology using R10 chemistry on their MinION platform.

### Whole-genome assembly

Whole-genome assembly for the Illumina-sequenced metagenomic data was performed using MetaWRAP [38] following read quality assessment and genome assembly using SPAdes v3.13 (metagenomic mode) [39]. Genomes were binned using an ensemble approach using Concoct [40], MaxBin2 [41], MetaBat2 [42] and CheckM v1.1.3 [43]. Nanopore-based whole-genome assembly was carried out using metaFlye v2.9.3 [44], with input specified as nanopore (--nano-hq) and minimum overlap (--min-overlap 1000), and the resulting assembly binned using MaxBin2, MetaBat2 and MetaDecoder [45]. The resulting genomes were polished with Pilon v1.24 [46] using paired Illumina data from the same *AFA* strains mapped using Bowtie2 v2.3.4.1 [47].

### Annotation, bioinformatics and statistical analysis

*Nostocales* genome sequences were downloaded from NCBI RefSeq (*n*=161). These, together with the assembled sequences obtained as described above, were all annotated using Prokka v1.14.6 [48] to ensure a consistent annotation across all genomes. The annotated genomes were then visualized using the Sanger Institute software Artemis [49]. Importantly, all bioinformatic analysis carried out on the CCAP 1401/3 and Klamath *AFA* was based on the Oxford Nanopore whole-genome sequences, whereas for NIES 4052 *AFA*, the Illumina sequence was used. Oxford Nanopore sequencing was not performed for NIES 4052 due to DNA availability and sequencing resource constraints at the time of analysis. CCAP 1401/3 and Klamath *AFA* Illumina sequences were used to investigate the module completeness for cobalamin production in the cyanobacteria and commensal bacteria. The latter was achieved by running the software MicrobeAnnotator to predict metabolic pathway completeness using Kyoto Encyclopedia of Genes and Genomes (KEGG)/KoFam pathway databases [50].

The following bioinformatic analyses were conducted on all 164 genomes. Genome quality and metrics were assessed using CheckM v1.1.3 [43]. One-way ANOVA was further used to calculate the statistical significance (*P*-value<0.05) of genomic statistical parameters between genera of the 164 genomes using routines in the SciPy package [51]. The Prokka annotated coding sequences were annotated using eggNOG-mapper v2.1.9 and database v5.0 (File S1, available in the online Supplementary Material) [52]. Protein domains were identified using pfam_scan v1.6 and Pfam 23.0 database (File S1) [53]. KEGG orthology assignments were performed using Kofamscan v1.3.0 (File S1) [54]. AntiSMASH v6.1.1 was used for the analysis and identification of BGCs within microbial genomes using the default detection parameters [55].

### Phylogenetic analysis

Phylogenetic reconstruction of all 164 genomes was performed using PhyloPhlAn v3.0.60 with the PhyloPhlAn 400 universal marker gene database [56]. Phylogenetic inference of the concatenated alignment was carried out using RAxML v8.2.12 [57], with 1,000 bootstrap replicates used to assess node support. The resulting tree was visualized using FigTree v1.4.4, and the complete phylogenetic tree is provided in Fig. S1.

To complement the marker-gene phylogeny with an independent genome-based taxonomic framework, all 164 genomes were classified using GTDB-Tk v2.3.2 with database release 214 [58] (Table S2b). GTDB-Tk classifications were used to assess genome-based taxonomic placement and to identify discordance between conventional NCBI/traditional nomenclature and the Genome Taxonomy Database (GTDB) framework. We acknowledge that GTDB does not recognize *Nostocales* as a formal order and instead places the analysed taxa within *Cyanobacteriales*. However, for consistency with the existing literature, accession records and the nomenclature used throughout the manuscript, organism names and higher-level taxonomic labels in the main text and figures are reported primarily in accordance with NCBI/traditional taxonomy, and GTDB-Tk classifications are used as supporting genome-based taxonomic evidence.

ANI analysis (fastANI v1.3.3 [59] was performed for the *AFA/ADA* clade, comprising *AFA*-labelled genomes and closely related *Aphanizomenon*, *Dolichospermum* and *Anabaena* genomes, to further evaluate the placement of strains highlighted as potentially misclassified in the phylogenomic tree. Pairwise ANI values were interpreted using the commonly applied ≥95% species-level threshold.

### Heatmap, gene and operon analysis

All genomes (.faa) were searched for cyanotoxin, natural competence, restriction enzyme and *btu* genes against reference sequences specified in the respective table and figure legends using blastp [60] implemented through a loop command in Ubuntu (Tables S3b–S6b and S8b–S11). As a reference-based homology approach, this analysis is most sensitive to genes closely related to the selected query sequences but may not detect more divergent homologues.

Cyanotoxin-associated biosynthetic genes were identified by blastp searches against reference toxin gene sets, as defined in Tables S3–S6. For each query, the top-scoring hit was retained and the percentage identity, e-value, bitscore, alignment length and peptide sequence were recorded. Hits were filtered using a maximum e-value threshold of 1e-20. Average percentage similarity across each reference gene set was calculated as a preliminary measure of overall homology. Putative cyanotoxin-associated BGCs were subsequently evaluated based on the presence of multiple expected biosynthetic genes and manual inspection of local gene organization relative to reference clusters. Where multiple copies of a gene were detected, the longest alignment was prioritized, followed by the lowest e-value, and redundant copies were pruned. Candidate BGCs were evaluated through the presence of multiple expected biosynthetic genes and inspection of local gene organization relative to known toxin operon architectures, using Artemis and antiSMASH outputs.

*Btu* transport genes were identified by blastp searches against using the *btuB, btuC, btuD* and *btuF* sequences from Klamath *AFA* as reference genes, as defined in Table S11. Hits were filtered using a maximum e-value threshold of 1e-20, and percentage identity, e-value, bitscore, alignment length and peptide sequence were recorded. Where multiple hits for the same gene were detected, the longest alignment was prioritized, followed by the lowest e-value, and redundant hits were removed. Average percentage similarity across the *btu* reference gene set was calculated as a comparative homology overview, rather than as standalone evidence of functional B_12_ transport. Putative *btu* operon organization was assessed through gene presence, order and local genomic context in the annotated genomes.

Putative R–M system-associated proteins were identified from Prokka annotations. To assess the reliability of these assignments, all 15 putative R–M system-associated proteins identified in the three newly sequenced *AFA* genomes were cross-validated against REBASE. Because annotation-based detection may overlook novel R–M systems or assign related proteins to broad R–M system-associated categories, these predictions were considered putative.

RNA regulatory elements, including cobalamin-related riboswitches, were identified using Infernal v1.1 [61] searches against Rfam covariance models [62] as part of Prokka annotation. The three newly sequenced *AFA* genomes were additionally re-annotated with Bakta v1.12 [63] as a complementary check for RNA regulatory regions. Putative cobalamin, AdoCbl and related RNA regulatory elements were summarized across the dataset and visualized alongside the *AFA/ADA* phylogeny.

### Measurement of vitamin B_12_ content in *AFA*

For measurement of B_12_ (cobalamin) and pseudo-B_12_ (pseudocobalamin), three 100 ml CCAP 1401/3 and three 100 ml NIES 4052 *AFA* cultures were grown to late exponential phase (OD_750_:0.8) in JM medium (without the addition of cobalt) using a 16 h:8 h L/D cycle, an irradiance of 50 µmol photons m^−2^ s^−1^, a temperature of 20 °C and a mixing rate of 80 r.p.m. The resulting cultures were centrifuged and washed (2,800 ***g*** for 5 min) three times, and the resulting pellets were lyophilized at −20 °C for 24 h. Due to the substantially larger biomass requirements of the assay relative to what was feasible from laboratory cultivation during the study period, Klamath Lake *AFA* biomass used for the vitamin B_12_ measurements was sourced commercially from the Italian *AFA* nutritional supplement company Blue Lotus s.r.l. As commercially processed biomass may introduce additional variability relative to laboratory-cultivated material, the analysis was intended primarily to determine the presence of active cobalamin forms within commercially representative Klamath *AFA* biomass rather than for strict quantitative comparison between strains.

For each sample, 3-5 mg of dried biomass was resuspended in 1 ml deionised water water and cobalamins were extracted by boiling for 20 min. The identities of the extracted cobalamins were then determined using the B_12_-dependent *Chlamydomonas reinhardtii* metE7 mutant assay, as described in Bunbury *et al*. [64]. This mutant lacks the B_12_-independent methionine synthase METE and therefore grows only when supplied with an active cobalamin, enabling functional discrimination of B_12_ forms.

### Uptake of a fluorescent B_12_ analogue

To investigate uptake and intracellular accumulation of cobalamin analogues in *AFA* strains, fluorescence-based uptake experiments were performed using the fluorescent vitamin B_12_ analogue BODIPY-TR-B_12_, hereafter (f)B_12_, with an excitation wavelength of 588 nm and an emission wavelength of 621 nm. Fluorescent analogues were supplied by the Warren Lab at the Quadram Institute, UK [65]. (f)B_12_ was added at a concentration of 1 µM to cultures of CCAP 1401/3, NIES 4052 and Klamath *AFA* in early exponential phase (OD_750_ : 0.3). Cultures were grown for a further 3 days in a 12-well plate (in triplicate) under a 16 h:8 h L/D cycle, an irradiance of 50 µmol photons m^−2^ s^−1^, a temperature of 20 °C at a mixing rate of 80 r.p.m. JM medium and JM + (f)B_12_ medium were used as a positive control, to ensure the fluorescence properties of (f)B_12_; *AFA* cultures without added (f)B_12_ were included as a negative control. BODIPY’s fluorescence was measured for each culture, its respective supernatant and pellet (*n*=3), both in a plate reader (FLUOstar Omega, BMG LABTECH, Germany) and confocal laser scanning microscope (MP_OPO SP8, Leica Microsystems, Germany) in two different experiments. The first experiment measured plate reader fluorescence on day 0 and plate reader and confocal fluorescence on day 3, whereas the second experiment measured plate reader fluorescence on days 0, 1, 2 and 3. Pellets were washed three times with fresh medium to eliminate any (f)B_12_ in the residual supernatant and maximize *AFA* abundance. BODIPY fluorescence was expressed relative to the endogenous fluorescence signal for phycocyanin (measured at 642 nm). For confocal analysis, a fluorescence threshold was applied to selectively capture *AFA* cells, identified by their endogenous phycocyanin fluorescence at 621 nm, while excluding contaminating commensal bacteria that lack phycocyanin fluorescence. This gating strategy was used to minimize the contribution of bacterial fluorescence to the *AFA*-associated (f)B_12_ signal. We further confirmed that the BODIPY emission signal (621 nm) did not excite phycocyanin (623 nm), which could lead to quenching of the BODIPY signal (data not shown). This protocol was based on that of [65].

## Results and discussion

### Genome statistics help identify atypical *AFA*-labelled genomes

Illumina WGS of *AFA* NIES 4052 produced 80 contigs with a completeness of 99.89%, a total assembly length of 4,398,211 nt, and a mean coverage of 94× (GCA_060050165.1). Oxford Nanopore sequencing of CCAP 1401/3 and Klamath *AFA* yielded two contigs (98.89% completeness) and one contig (98.44% completeness), respectively, indicating near-complete genome sequences without significant gaps. The CCAP 1401/3 (GCA_060050145.1) and Klamath *AFA* assemblies (GCA_060050125.1) had total lengths of 5,867,837 nt and 4,483,877 nt, with mean coverages of 119× and 68×, respectively. The second contig of CCAP 1401/3 (14,404 bp; 4612× coverage) represents a putative *Nostocales* plasmid (NCBI BioProject: PRJNA1207407). ORFs (*n*=13) were identified within the plasmid, of which three were distantly related to other *Nostocales* sequence data, and an additional manually assigned reading frame at the 5′ terminus had ca. 60% aa identity to a *Fisherella* sp. telomere resolvase, suggesting that this second contig is a linear plasmid.

**Fig. 1.**
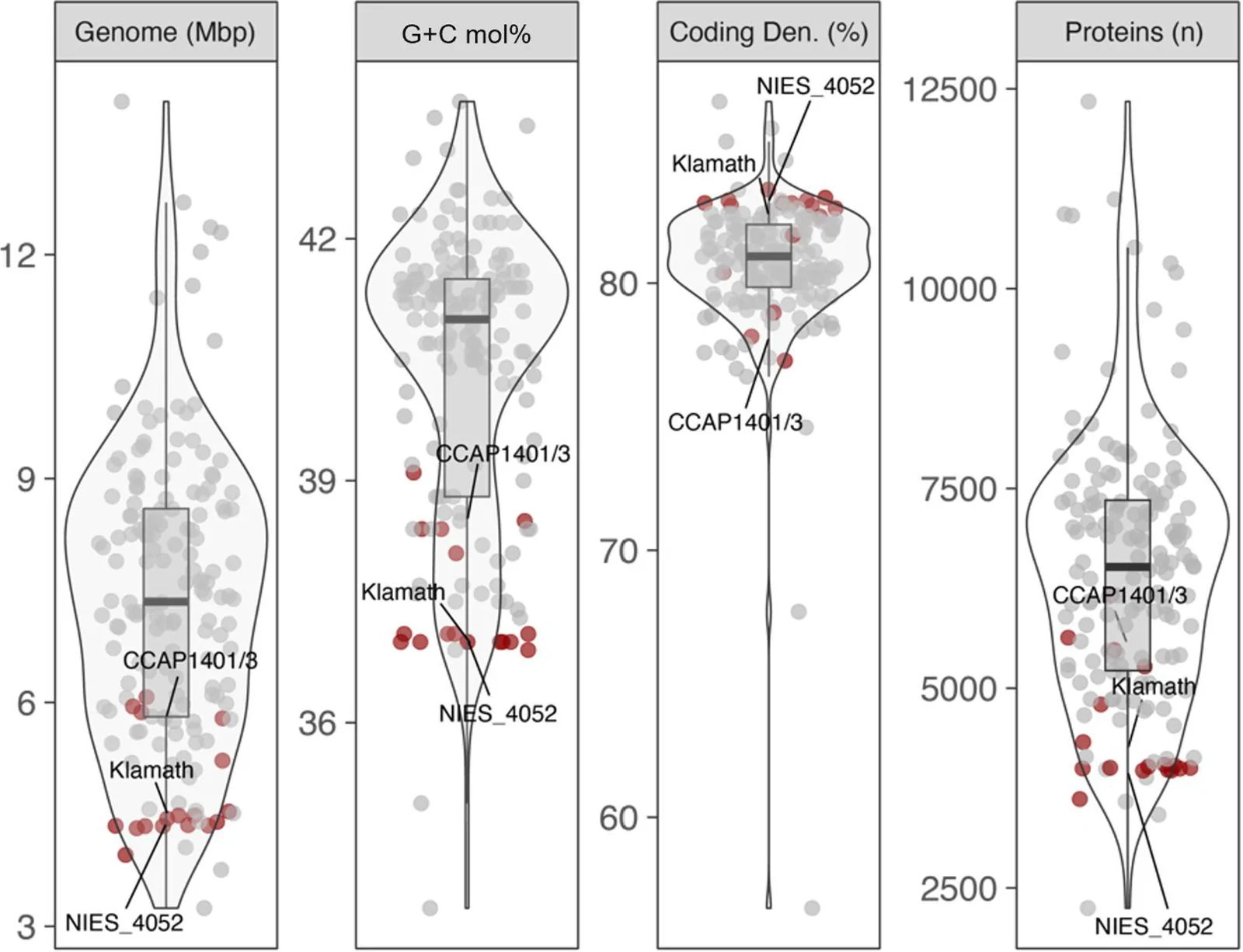
*Nostocales* assembled genome statistics. Genomes labelled as *AFA* are highlighted (red points), and the position of the three newly sequenced genomes in this study is shown. Data derived from Table S2a.

Analysis of 164 *Nostocales* genomes revealed genome sizes ranging from 3.2 to 14 Mb, with an average of 7.4 Mb (Table S2a). Coding density varied between 56.6 and 86.8%, with a mean of 80.6%, while the number of protein-coding genes ranged from 2,248 to 12,341, with a mean of 6,432. G+C content was found to be between 33.7 and 43.7 mol%, with an average of 40.3 mol% (Fig. 1). These values showed clear differences among genera, indicating substantial genomic variability within the order. A one-way ANOVA analysis revealed that G+C content (*P*<1.33×10^−25^), genome size (*P*<1.61×10^−21^), protein-coding genes (*P*<7.01×10^−19^) and coding density (*P*<3.01×10^−5^) are statistically significant parameters for *Nostocales* genus-level differentiation. While these metrics are not used for taxonomic classification, the statistical separation highlights consistent genus-associated trends across the dataset.

Table 1 highlights the genome statistics of the 16 genomes labelled as *AFA* in NCBI or associated records. *AFA* strains exhibited an average G+C content of 37 mol%, an average genome size of 4.36 Mb, 3,995 protein-coding genes and an average coding density of 83%. Comparative genome-statistics analysis identified four *AFA*-labelled genomes as atypical relative to the main *AFA* group: namely, CCAP 1401/3, FACHB-1040, KM1D3_PB and WA102 (Table 1). Notably, CCAP 1401/3 has recently undergone taxonomic reclassification based on morphological assessments, from *AFA* to *Aphanizomenon klebahnii* (https://www.ccap.ac.uk/catalogue/strain-1401-3). Similarly, KM1D3_PB is an atypical *AFA* strain, as it inhabits the Baltic Sea, a brackish environment, making it the only known *AFA* strain not to reside in a freshwater lake [66]. Published taxonomic information is limited for FACHB-1040 and WA102, and their status is therefore evaluated further using phylogenomic and ANI analyses.

**Table 1.** Genome statistics of the 16 *AFA*-labelled genomes analysed within this study. The genomes CCAP 1401/3, Klamath and NIES 4052 were sequenced in this study, while the others were downloaded from NCBI. The genomes CCAP 1401/3, FACHB-1040, KM1D3_PB and WA102 are potentially incorrectly described as *AFA* based on the genome statistics shown in bold. Genome statistics were determined using CheckM (version 1).

Organism name	Comp. (%)	Genome size	Cod. den.	Prot-coding	G+C (mol%)	Con.	Scaff.	N50 con.	N50 scaff.
CCAP 1401/3	98.89	**5,867,837**	**78**	**5,478**	**38.5**	2	2	5,867,837	5,867,837
Clear-A1	99.89	4,442,226	83.1	4,003	37.1	85	60	97,558	120,880
CP01	99.22	4,356,503	83.2	4,004	36.9	152	152	54,035	54,035
DEX188	99.89	4,538,078	83.5	4,042	37.1	1	1	4,538,078	4,538,078
FACHB-1040	99.22	**5,786,747**	**78.9**	**5,271**	**38.4**	155	155	58,342	58,342
FACHB-1171	99.44	4,343,146	82.9	3,995	37	91	91	78,897	78,897
FACHB-1249	99.89	4,350,034	82.8	4,020	37	97	97	83,039	83,039
FACHB-1265	99.89	4,343,226	82.9	3,976	37	88	88	85,704	85,704
FACHB-1287	99.89	4,347,270	83	3,994	37	85	85	85,728	85,728
FACHB-1290	99.67	4,314,391	83	3,969	37	136	136	55,341	55,341
Klamath	98.44	4,483,877	82.5	4,326	37.1	1	1	4,483,877	4,483,877
KM1D3_PB	99.44	**6,071,322**	**77.1**	**5,633**	**38.4**	1	1	6,071,322	6,071,322
NIES 4052	99.89	4,398,211	83	3,969	37.1	80	68	124,253	157,781
NRERC-008	85.84	3,957,023	n/a	3,613	37	56	56	101,067	101,067
UKL13-PB	99.67	4,484,012	83.1	4,032	37	15	15	767,568	767,568
WA102	99.89	**5,946,039**	**80.4**	**6,165**	**39.1**	116	1,160	24,228	24,228

Cod. Den., coding density; Comp, completeness; Con., contigs; G+C mol%, guanine–cytosine content; N50 Con., N50 contig length; N50 Scaff., N50 scaffold length; Prot-coding, protein-coding genes; Scaff., scaffolds.

Altogether, these genome statistics provide a useful preliminary framework for identifying atypical *AFA*-labelled genomes. However, these metrics are not sufficient for taxonomic reassignment on their own and are therefore interpreted here as complementary to the phylogenomic, GTDB-Tk and ANI analyses described below.

### Phylogenomic analysis supports reassessment of *AFA*-labelled genomes

**Fig. 2.**
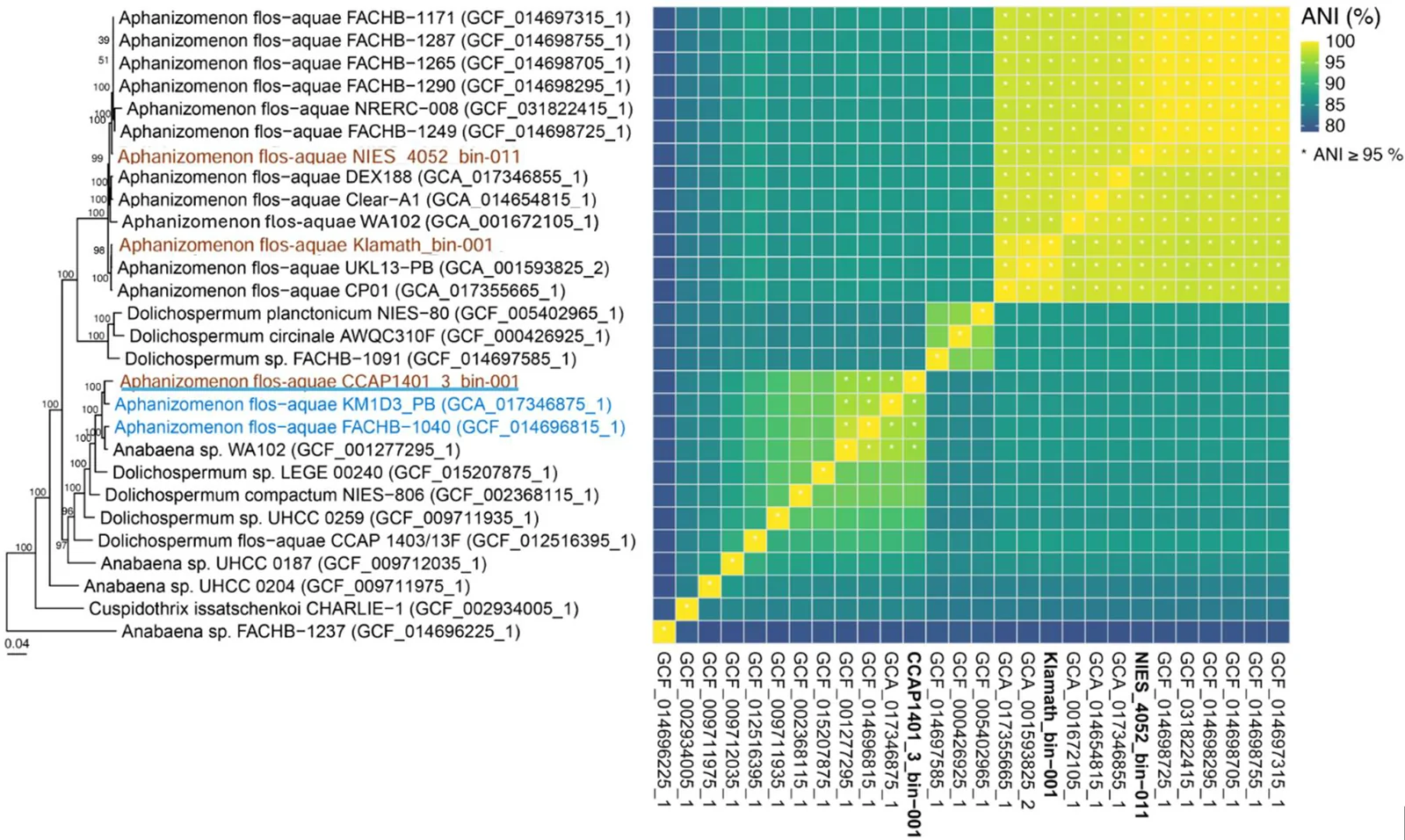
Maximum-likelihood phylogenetic tree and pairwise ANI heatmap of *AFA*-labelled genomes and closely related *Dolichospermum* and *Anabaena* strains, corresponding to the *ADA* clade described by Dreher *et al*. [12]. This figure shows an enlarged view from the full 164-genome phylogeny shown in Fig. S1. The tree was reconstructed with PhyloPhlAn v3.0.60 and RAxML v8.2.12 using 1,000 bootstrap replicates. Genomes sequenced in this study are shown in red, whereas genomes in blue represent *AFA*-labelled strains requiring taxonomic reassessment. CCAP 1401/3 *AFA* is underscored in blue as it is both newly sequenced and may require taxonomic reassessment. Pairwise ANI values are shown as a heatmap, with asterisks indicating ANI≥95%, the intra-species threshold.

A pangenome phylogenetic tree was constructed for the 164 *Nostocales* genomes (Fig. S1), with a subset of the tree focusing on the *AFA-*labelled genomes and the closely related *Anabaena* and *Dolichospermum* genomes shown in Fig. 2. The tree supports the conclusions drawn from the genome statistics (Table 1), showing that FACHB-1040, KM1D3_PB and CCAP 1401/3 fall outside the core *AFA*-associated cluster, supporting their reassessment as atypical or potentially misclassified *AFA*-labelled genomes. CCAP 1401/3 has now been officially recognized as a strain of *Aph. klebahnii* based on morphological assessment (https://www.ccap.ac.uk/catalogue/strain-1401-3).

To strengthen this interpretation, GTDB-Tk classification and pairwise ANI analysis was incorporated alongside the phylogenomic tree (Table S2b). Pairwise ANI values supported the separation of CCAP 1401/3, FACHB-1040 and KM1D3_PB from Klamath *AFA*, with ANI values of 86.58, 86.95 and 86.69%, respectively, which are all below the commonly applied 95% species-level threshold [59]. In contrast, although WA102 was atypical based on genome statistics, including a larger genome size and higher G+C content, it shared 97.77% ANI with Klamath *AFA*, above the species-level threshold. Therefore, WA102 is retained within the *AFA*-associated species-level group on the basis of ANI analysis. These findings highlight that genome statistics are useful initial screening metrics for identifying atypical *AFA* strains, but should not be treated as definitive taxonomic evidence without phylogenomic and ANI support.

A geographic signature was observed within the phylogenetic affiliations of the *AFA* species complex. *AFA* CP01, UKL13-PB and Klamath *AFA* all originate from Oregon, USA, with UKL13-PB and Klamath *AFA* being particularly closely related (ANI=99.6%). *AFA* WA102, Clear-A1 and DEX188 originate from Oregon and California, whereas the FACHB, NIES and NRERC strains originated from China, Japan and South Korea, respectively [9, 12, 18]. Together, these results support a more restricted interpretation of the *AFA* species-level cluster within the broader *Aphanizomenon* genus and the wider *ADA* clade, and highlight the need for caution when relying on historical NCBI species assignments [2].

### Putative cyanotoxin-associated BGCs are detected in some *Nostocales* genomes, but not in *AFA* strains

The genome sequences of all 164 *Nostocales* were searched for homologues of biosynthesis genes for known cyanotoxins: namely, ATX-a, SXT, MYCs/NODs and CYR (Table 2). ATX is a neurotoxin and acts as an agonist for nicotinic acetylcholine receptors, causing overstimulation of neurons, which leads to muscle twitching, paralysis and convulsions [13, 67]. SXT is a neurotoxin classified as a paralytic shellfish toxin, known for its ability to block sodium channels in nerve cells, leading to paralysis and, in severe cases, respiratory failure [68, 69]. MYCs and NODs are hepatotoxins which primarily target liver cells by inhibiting protein phosphatases (PP1 and PP2A), leading to liver damage and, in high concentrations, liver failure [70]. CYR is a tricyclic alkaloid toxin with hepatotoxic, cytotoxic and potentially carcinogenic effects [71].

**Table 2.** Presumptive cyanotoxin-associated biosynthetic gene detection in *Nostocales* genomes. blastp was used to query each genome for homologues of biosynthetic genes associated with ATX, SXT, CYR, MYC and NOD. Values represent the average top-hit percentage identity across the corresponding reference gene set and are presented as a preliminary homology summary only. ATX searches used *anaA–anaH from Oscillatoria* sp. PCC 6506; SXT searches used *sxtA–sxtE*, *sxtG–sxtN*, *sxtR–sxtT* and *sxtQ–sxtW* from *Dolichospermum circinale* AWQC131C; CYR searches used *cyrA–cyrK from Aphanizomenon* sp. 10E6; MYC/NOD searches used *mycA–mycJ* from *Microcystis aeruginosa* PCC 7806 [117, 118]. MYC reference genes were also used to screen for putative NOD-associated clusters because of the similarity between *mcy* and *nda* genes. blastp hits were filtered using a maximum e-value threshold of 1e-20. Where multiple hits for the same gene were detected, the longest alignment was prioritized, followed by the lowest e-value, and redundant hits were removed. No additional fixed percentage identity or coverage threshold was applied. Positive cyanotoxin BGC assignments were not based on average percentage identity alone, but on the complete gene-level blastp outputs, including percentage identity, e-value, bitscore and alignment length, reported in Tables S3b–S6b, together with manual inspection of local gene organization relative to the reference clusters shown in Fig. 3 and cross-comparison with the antiSMASH outputs summarized in Table S7

Organism name	ANA (%)	SXT (%)	CYR (%)	MC/ND (%)	Organism name	ANA (%)	SXT (%)	CYR (%)	MC/ND (%)	Legend
										90–100
*Aet. hydrillicola* CCALA 1050	38.7		36.3	36.1	*Nod.* sp. LEGE 06071	30.6		24.8	37.0	80–90
*Ama. nigriterrae* CENA67	31.1		32.8	36.8	*Nod.* sp. NIES-3585	44.1	21.2	25.2	39.2	70–80
*Ana. azotica* FACHB-119	40.8		28.4	41.3	*Nod. sphaerocarpa* UHCC 0038	31.8	20.8	29.2	40.1	60–70
*Ana. catenula* FACHB-362	34.7		23.5	35.6	*Nod. spumigena* CCY9414	37.6		26.5	68.3	50–60
*Ana. cylindrica* PCC 7122	42.0		32.7	35.5	*Nos. azollae* 0708	32.0			29.5	40–50
*Ana. lutea* FACHB-196	32.6		25.1	35.7	*Nos. calcicola* FACHB-3891	36.4		31.8	39.0	30–40
*Ana. minutissima* FACHB-250	35.1		31.7	38.8	*Nos.* cf*. commune* SO-36	40.0		26.4	36.9	20–30
*Ana.* sp. 4–3	32.2		27.7	38.8	*Nos.* cf*. edaphicum* LEGE 07299	32.9		25.4	37.7	0
*Ana.* sp. FACHB-1237	36.5		29.1	36.9	*Nos. commune* NIES-4072	40.8		31.7	38.4	
*Ana.* sp. PCC 7108	36.7		32.1	34.9	*Nos. edaphicum* CCNP1411	32.0		28.2	38.8	
*Ana.* sp. UHCC 0187	31.9		32.5	32.3	*Nos. favosum* CHAB5714	40.6		26.7	36.0	
*Ana.* sp. UHCC 0204	31.0		29.8	36.9	*Nos. flagelliforme* CCNUN1	40.6		32.7	35.6	
*Ana.* sp. WA102	81.2	25.3	36.0	34.3	*Nos. flagelliforme* FACHB-838	38.7		33.0	40.3	
*Ana. sphaerica* FACHB-251	37.4		30.3	35.0	*Nos. foliaceum* FACHB-393	41.0		35.7	39.0	
*Ana. subtropica* FACHB-260	34.8		28.5	36.3	*Nos. linckia* FACHB-104	40.2		27.0	38.3	
*Ana. elenkinii* CCIBt3563			23.1	28.2	*Nos. linckia* NIES-25	35.7		30.2	38.0	
***Aph. flos-aquae* CCAP 1401/3**	34.2		28.6	38.0	*Nos. linckia* z4	34.3	20.2	28.1	34.7	
*Aph. flos-aquae* Clear-A1	20.2		25.2	31.8	*Nos. mirabile* CHAB5784	43.5		30.5	35.6	
*Aph. flos-aquae* CP01	20.3		25.4	32.5	*Nos. paludosum* FACHB-159	37.3		32.6	33.7	
*Aph. flos-aquae* DEX188	20.3		23.6	32.3	*Nos. parmelioides* FACHB-3921	31.6		26.5	39.7	
*Aph. flos-aquae* FACHB-1040	80.8		31.4	37.0	*Nos. piscinale* CENA21	42.8		33.0	38.5	
*Aph. flos-aquae* FACHB-1171	20.5			32.4	*Nos. punctiforme* PCC 73102	31.2		32.2	39.0	
*Aph. flos-aquae* FACHB-1249	20.5			32.4	*Nos.* sp. 106C	34.8		27.3	34.7	
*Aph. flos-aquae* FACHB-1265	20.5			32.4	*Nos.* sp. 210A	26.4		25.7	39.5	
*Aph. flos-aquae* FACHB-1287	20.5			32.4	*Nos.* sp. 5183	44.7		22.7	40.7	
*Aph. flos-aquae* FACHB-1290	20.5			32.4	*Nos.* sp. B(2019)	35.0	28.3	34.2	39.5	
***Aph. flos-aquae* Klamath**	20.3		25.4	32.6	*Nos.* sp. C052	44.9		31.4	39.2	
*Aph. flos-aquae* KM1D3_PB	33.2		28.9	34.6	*Nos.* sp. CENA543	25.9		27.7	68.1	
***Aph. flos-aquae* NIES 4052**	24.5		23.7	32.4	*Nos.* sp. CHAB 5715	38.2		28.9	38.5	
*Aph. flos-aquae* NRERC-008				32.2	*Nos.* sp. CHAB 5824	35.2		21.6	38.9	
*Aph. flos-aquae* UKL13-PB	20.3		25.0	32.5	*Nos.* sp. CHAB 5836	36.8		24.6	71.9	
*Aph. flos-aquae* WA102	26.2		23.4	33.2	*Nos.* sp. CHAB 5844	30.5		29.0	36.9	
*Atl. silvestris* CENA357	42.1		31.1	36.3	*Nos.* sp. CMAA1605	31.6		31.4	71.4	
*Aul.* sp. FACHB-615	34.8		27.6	37.6	*Nos.* sp. FACHB-110	33.3		33.6	39.7	
*Bra. bromeliae* SPC951	28.7		28.1	33.9	*Nos.* sp. FACHB-152	40.0	28.4	31.4	37.9	
*Bra. sennae* CENA114	37.0		34.0	39.2	*Nos.* sp. FACHB-280	34.5		29.3	38.0	
*Bra.* sp. CT11	36.9		35.8	42.6	*Nos.* sp. FACHB-892	40.5		31.7	38.0	
*Bra.* sp. UFV-L1	41.1		32.6	37.4	*Nos.* sp. FACHB-973	39.1		32.8	39.2	
*Cal. anomala* FACHB-343	40.0		27.4	43.1	*Nos.* sp. KVJ20	32.8	25.4	30.3	38.5	
*Cal. brevissima* NIES-22	41.9		27.8	37.3	*Nos.* sp. LEGE 06077	28.2		28.9	38.1	
*Cal. desertica* PCC 7102	38.6		36.7	38.2	*Nos.* sp. LEGE 12450	30.5	26.5	27.2	39.8	
*Cal. elsteri* CCALA 953	25.1		27.6	32.8	*Nos.* sp. MG11	35.6	29.4	37.9	48.0	
*Cal. membranacea* FACHB-236	30.7		23.8	35.9	*Nos.* sp. MS1	28.4		33.0	39.7	
*Cal. parasitica* NIES-267	41.2		33.3	35.0	*Nos.* sp. N6	49.6	26.9	25.9	39.5	
*Cal. rhizosoleniae* SC01	36.9		32.8	33.5	*Nos.* sp. NIES-3756	28.4		29.4	37.8	
*Cal.* sp. 336/3	22.2		23.0	34.5	*Nos.* sp. NIES-4103	29.6		30.1	37.8	
*Cal.* sp. HK-06	39.8		34.5	36.0	*Nos.* sp. PA-18–2419	35.0			36.6	
*Cal.* sp. NIES-2098	22.2		29.6	34.0	*Nos.* sp. PCC 7107	34.3		28.9	35.1	
*Cal.* sp. NIES-2100	37.0		33.0	38.4	*Nos.* sp. PCC 7120	31.4		24.4	36.1	
*Cal.* sp. NIES-3974			22.7	27.1	*Nos.* sp. PCC 7524	28.0		23.6	31.8	
*Cal.* sp. NIES-4105	38.8		35.2	44.4	*Nos.* sp. T09	33.2		26.4	33.4	
*Cal.* sp. PCC 6303	31.0		23.6	34.8	*Nos.* sp. TCL240-02	38.9		25.4	39.5	
*Cal.* sp. PCC 7103	38.9		33.9	35.0	*Nos.* sp. TCL26-01	22.7		24.0	33.1	
*Cal.* sp. PCC 7507	45.0		23.1	33.9	*Nos.* sp. UCD121	31.4		31.7	40.3	
*Cal.* sp. PCC 7716	46.8	21.0	36.4	36.8	*Nos.* sp. UHCC 0702	32.2		32.5	39.3	
*Chl. fritschii* PCC 6912	31.7		26.1	37.5	*Nos.* sp. UHCC 0870	37.9		29.5	39.7	
*Cus. issatschenkoi* CHARLIE-1	83.1		34.6	33.5	*Nos.* sp. WHI	39.9		25.8	64.3	
*Cyl. curvispora* GIHE-G1	21.1		24.6	30.9	*Nos.* sp. XA010	36.2		29.5	39.1	
*Cyl. raciborskii* Cr2010	28.0		23.5	31.2	*Nos. sphaeroides* Kutzing	24.5		22.1	32.8	
*Cyl.* sp. FACHB-282	32.2		27.8	39.2	*Nos. spongiaeforme* FACHB-130	34.6		28.9	36.0	
*Cyl. stagnale* PCC 7417	84.0		32.7	38.3	*Ple.* cf*. radiosum* LEGE 06105	31.7		30.1	36.3	
*Den. phyllosphericum* CENA369	44.2		32.7	40.5	*Ric. intracellularis* HH01				26.4	
*Des. muscorum* FACHB-395	31.8		32.7	39.9	*Ric. sinica* FACHB-800	28.1		26.2	34.4	
*Dol. circinale* AWQC310F	29.8			32.8	*Riv.* sp. PCC 7116	37.8		38.0	35.5	
*Dol. compactum* NIES-806	27.7		24.3	37.2	*Roh.* sp. LEGE 12411	41.1	25.2	38.2	43.9	
*Dol. flos-aquae* CCAP 1403/13F	37.6		28.7	42.9	*Scy. hofmanni* UTEX2349	45.4	31.1	38.2	39.6	
*Dol. planctonicum* NIES-80	20.5			28.7	*Scy. hofmannii* PCC 7110	45.4	20.2	34.9	41.9	
*Dol.* sp. FACHB-1091	26.6			33.0	*Scy.* sp. HK-05	39.0		35.1	37.5	
*Dol.* sp. LEGE 00240	38.0	94.9	38.2	35.4	*Scy.* sp. NIES-4073	38.2	29.4	28.8	47.2	
*Dol.* sp. UHCC 0259	32.1		24.5	33.3	*Scy.* sp. UIC 10036	45.4		30.6	40.0	
*Fis. muscicola* PCC 7414	29.5		24.0	33.3	*Scy. tolypothrichoides* VB-61278	34.1		34.6	36.8	
*Fis. muscicola* SAG 1427–1	42.4		25.3	34.8	*Sph. aphanizomenoides* BCCUSP55	38.1		30.5	33.8	
*Fis.* sp. PCC 9431	37.1		30.2	41.1	*Sph. kisseleviana* NIES-73	27.8			35.5	
*Fis.* sp. PCC 9605	46.2		32.0	38.1	*Sph. torques-reginae* ITEP-024	34.9		27.0	36.7	
*Fis. thermalis* PCC 7521	30.1		23.7	33.9	*Tol. bouteillei* VB521301	41.2		30.5	41.4	
*For. contorta* PCC 7126	29.8		28.0	34.0	*Tol.* sp. FACHB-123	39.2		23.2	35.2	
*For.* sp. LEGE XX443	26.7		28.0	34.8	*Tol.* sp. NIES-4075	33.2	26.9	32.3	44.1	
*Has. byssoidea* VB512170	29.2		28.4	34.8	*Tol.* sp. PCC 7910			23.7	38.5	
*Ini. tapete* BLCC-T55	42.3		36.8	67.5	*Tol. tenuis* PCC 7101	33.7		27.5	36.6	
*Kom.* sp*. ‘*clone 1’	29.0	27.6	36.3	47.2	*Tri.* sp. NMC-1	31.1		25.8	33.2	
*Mas. repens* PCC 10914	35.2		23.6	35.0	*Tri. variabilis* 0441	40.3		27.4	38.3	
*Mas. testarum* BC008	36.0	12.4	34.3	39.8	*Wes. prolifica* IICB1	29.6	16.4	33.7	70.0	

The corresponding full genus names are as follows: Aet. = Aetokthonos; Ama. = Amazonocrinis; Ana. = Anabaena; Aph. = Aphanizomenon; Atl. = Atlanticothrix; Aul. = Aulosira; Bra. = Brasilonema; Cal. = Calothrix; Chl. = Chlorogloeopsis; Cus. = Cuspidothrix; Cyl. = Cylindrospermopsis; Den. = Dendronalium; Des. = Desmonostoc; Dol. = Dolichospermum; Fis. = Fischerella; For. = Fortiea; Has. = Hassallia; Ini. = Iningainema; Kom. = Komarekiella; Mas. = Mastigocladopsis; Nod. = Nodularia; Nos. = Nostoc; Ple. = Plectonema; Ric. = Richelia; Riv. = Rivularia; Roh. = Roholtiella; Scy. = Scytonema; Sph. = Sphaerospermopsis; Tol. = Tolypothrix; Tri. = Trichormus; Wes. = Westellopsis.

**Fig. 3.**
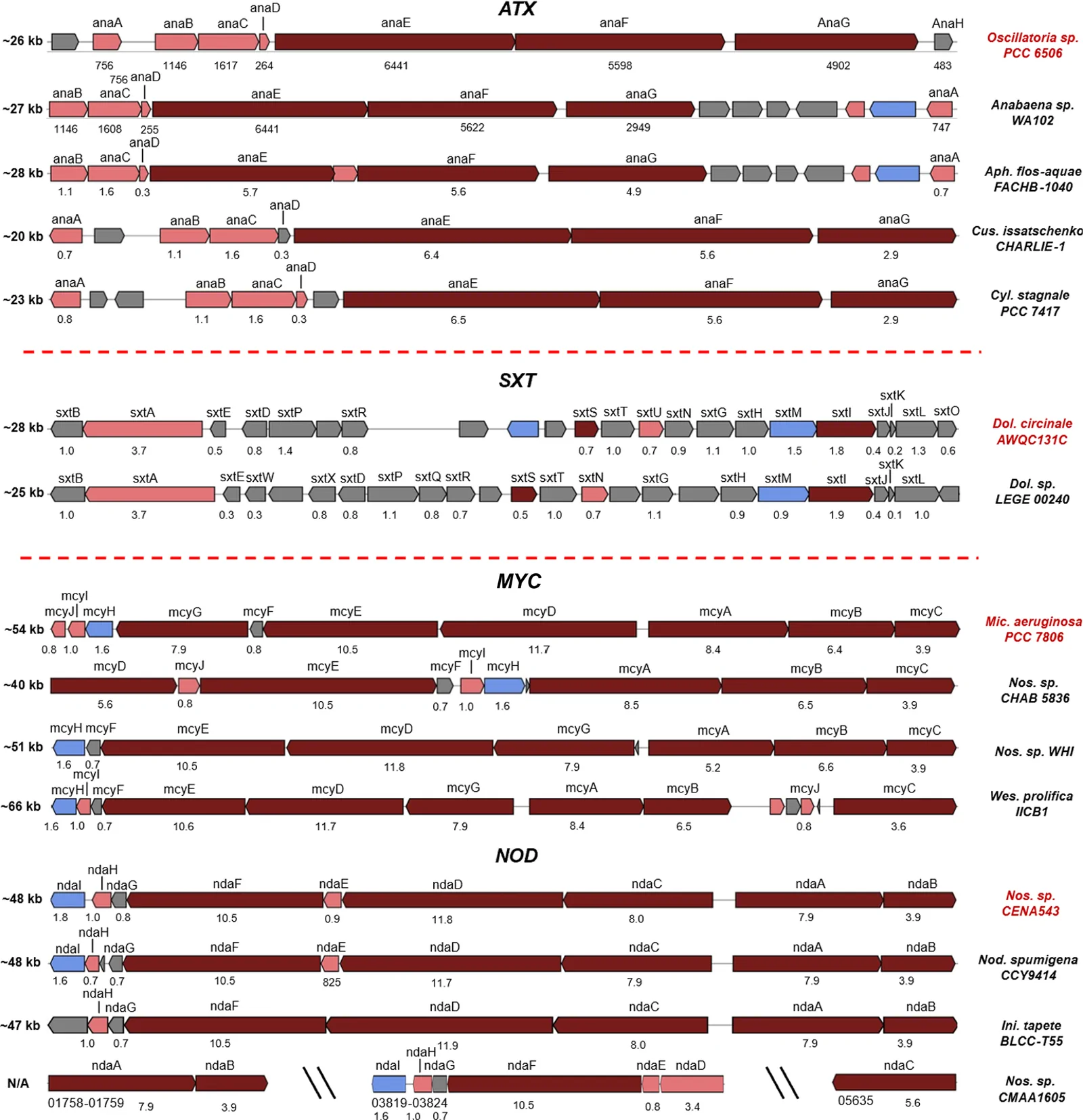
Putative cyanotoxin-associated BGCs for ATX, SXT, MYC and NOD in *Nostocales* strains identified in Table 2, compared with reference cyanotoxin biosynthetic operons obtained from MIBiG. ATX: *Oscillatoria* sp. PCC 6506 [76]; SXT: *Dol. circinale* AWQC131C [77]; MYC: *Mic. aeruginosa* PCC 7806 [118]; NOD: *Nos.* sp. CENA543 [83]. The latter was also included within the analysed dataset. Operons are not drawn to a common scale across organisms, but genes within each operon are drawn approximately to scale. Gene lengths are shown below each coding sequence as approximate kilobase values, rounded to the nearest 0.1 kb. Gene colours were referenced from antiSMASH and MIBiG: burgundy, core biosynthetic genes; pastel red, additional biosynthetic genes; pastel blue, transport-related genes; grey, other genes. Candidate BGCs shown in this figure were manually inspected for gene organization relative to MIBiG reference clusters using Artemis and cross-compared with antiSMASH outputs summarized in Table S7.

Four genomes out of the 164 analysed were identified as containing putative ATX-associated BGCs consistent with ATX/ATX-analogue biosynthesis: *Ana.* sp. WA102, *Cus. issatschenkoi* CHARLIE-1, *Cylindrospermopsis stagnale* PCC 7417 and *AFA* FACHB-1040 (Table 2, Fig. 3, Tables S3a and S3b). The first three organisms are confirmed ATX producers, with *Cyl. stagnale* PCC 7417 also producing dihydroATX [72, 73]. Notably, *AFA* FACHB-1040 also contained a putative ATX-associated cluster, although toxin production requires experimental verification. The key biosynthetic genes identified include *anaA*, *anaC* and *anaF* [74, 75]. Genomic variations, such as translocation of the *anaA* gene, may influence ATX production and the synthesis of analogues such as homo-ATX [76]. This translocation is observed in the *ana* operon of *Osc.* sp. PCC 6506 (Fig. 3) that is known to produce homo-ATX, while *Ana.* sp. WA102 produces ATX [72].

**Table 3.** *btu* operon analysis within 164 *Nostocales* genomes using the Klamath *AFA btu* genes, *btuB*, *btuC*, *btuF* and *btuD*, as reference sequences (Table S11). Values represent the percentage sequence similarity to the reference sequence. Grey cells indicate that no match has been found. Operon descriptions: ‘Yes’ indicates the same *btu* operon arrangement as observed in Klamath *AFA* (*btuB–frdA–btuF–btuC–btuD*), ‘Yes*’ indicates that the genome in question has the same *btu* operon arrangement as Klamath *AFA* minus *btuD*, ‘No’ indicates that no operon was found and ‘No*’ indicates that a *btu* operon different from Klamath *AFA* may be found

Organism name	BtuB (%)	BtuC (%)	BtuD (%)	BtuF (%)	Operon	Organism name	BtuB (%)	BtuC (%)	BtuD (%)	BtuF (%)	Operon	Legend
*Aet. hydrillicola* CCALA 1050	39.7		32.9		No	*Nod.* sp. LEGE 06071	27.6	70.3	33.9	65.8	No*	90–100
*Ama. nigriterrae* CENA67	27.4	32.2	37.8		No	*Nod.* sp. NIES-3585	28.5	33.7	33.9		No	80–90
*Ana. azotica* FACHB-119	29.6	34.9	33.2		No	*Nod. sphaerocarpa* UHCC 0038	28.5	31.6	33.5		No	70–80
*Ana. catenula* FACHB-362	27.7	33.3	37.6	39.5	No	*Nod. spumigena* CCY9414	28.8	32.4	33.5	23.4	No	60–70
*Ana. cylindrica* PCC 7122	91.9	93.4	87.8	85.3	**Yes**	*Nos. azollae* 0708	33.3	39.1	29.1		No	50–60
*Ana. lutea* FACHB-196	28.2	33.6	37.8		No	*Nos. calcicola* FACHB-3891	77.1	83.9	73.7	69.4	**Yes**	40–50
*Ana. minutissima* FACHB-250	76.5	82.9	72.8	68.8	**Yes**	*Nos.* cf*. commune* SO-36	27.7	33.2	33.1		No	30–40
*Ana.* sp. 4–3	28.6	32.7	33.5	24.3	No	*Nos.* cf*. edaphicum* LEGE 07299	27.4		34.5		No	20–30
*Ana.* sp. FACHB-1237	29.0		32.4		No	*Nos. commune* NIES-4072	28.2	32.9	34.4		No	0
*Ana.* sp. PCC 7108	91.2	93.6	87.8	85.7	**Yes**	*Nos. edaphicum* CCNP1411	78.7	82.0	77.4	71.7	**Yes**	
*Ana.* sp. UHCC 0187	27.5		32.0		No	*Nos. favosum* CHAB5714	69.5	82.0	77.4	71.0	**Yes**	
*Ana.* sp. UHCC 0204	90.8	86.8	84.5	83.0	**Yes**	*Nos. flagelliforme* CCNUN1	27.9	33.1	38.2		No	
*Ana.* sp. WA102	91.1	87.3	87.5	86.8	**Yes**	*Nos. flagelliforme* FACHB-838	28.6	34.1	33.2		No	
*Ana. sphaerica* FACHB-251	28.7	32.0	33.9		No	*Nos. foliaceum* FACHB-393	27.3	33.5	36.1		No	
*Ana. subtropica* FACHB-260	27.8	32.9	33.9		No	*Nos. linckia* FACHB-104	28.6	32.5	32.1		No	
*Ana. elenkinii* CCIBt3563	69.7	73.0	36.3	63.0	Yes*	*Nos. linckia* NIES-25	28.7	33.7	35.7		No	
***Aph. flos-aquae* CCAP 1401/3**	29.1		31.2		No	*Nos. linckia* z4	75.6	82.3	71.1	68.9	**Yes**	
*Aph. flos-aquae* Clear-A1	98.9	99.7	100.0	99.5	**Yes**	*Nos. mirabile* CHAB5784	27.9	33.1	37.4		No	
*Aph. flos-aquae* CP01	99.4	99.7	100.0	99.8	**Yes**	*Nos. paludosum* FACHB-159	28.2	33.4	37.6		No	
*Aph. flos-aquae* DEX188	100.0	100.0	100.0	99.5	**Yes**	*Nos. parmelioides* FACHB-3921	75.9	81.2	70.6	69.0	**Yes**	
*Aph. flos-aquae* FACHB-1040	91.1	87.9	87.8	84.8	**Yes**	*Nos. piscinale* CENA21	27.6	33.3	33.9		No	
*Aph. flos-aquae* FACHB-1171	27.8		32.1		No	*Nos. punctiforme* PCC 73102	27.1	32.1	37.8		No	
*Aph. flos-aquae* FACHB-1249	27.8		32.1		No	*Nos.* sp. 106C	28.7	32.9	37.2		No	
*Aph. flos-aquae* FACHB-1265	27.8		32.1		No	*Nos.* sp. 210A	27.8	33.7	34.3		No	
*Aph. flos-aquae* FACHB-1287	27.8		32.1		No	*Nos.* sp. 5183	28.6	32.3	33.9		No	
*Aph. flos-aquae* FACHB-1290	27.8		32.1		No	*Nos.* sp. B(2019)	29.0	33.8	34.3	24.6	No	
***Aph. flos-aquae* Klamath**	100.0	100.0	100.0	100.0	**Yes**	*Nos.* sp. C052	27.6	33.2	33.6		No	
*Aph. flos-aquae* KM1D3_PB	91.6	87.9	87.8	85.3	**Yes**	*Nos.* sp. CENA543	28.2	33.3	34.4		No	
***Aph. flos-aquae* NIES 4052**	27.7		32.1		No	*Nos.* sp. CHAB 5715	77.3	35.1	74.7	68.0	No	
*Aph. flos-aquae* NRERC-008	27.8		32.1		No	*Nos.* sp. CHAB 5824	28.3	32.6	37.0		No	
*Aph. flos-aquae* UKL13-PB	100.0	100.0	100.0	99.8	**Yes**	*Nos.* sp. CHAB 5836	21.3	33.5	36.7		No	
*Aph. flos-aquae* WA102	28.1		31.4		No	*Nos.* sp. CHAB 5844	29.5	32.7	37.4		No	
*Atl. silvestris* CENA357	27.8	33.3	36.0		No	*Nos.* sp. CMAA1605	28.1	33.7	34.4		No	
*Aul.* sp. FACHB-615	28.7	32.7	33.1		No	*Nos.* sp. FACHB-110	28.7	32.3	34.3	21.7	No	
*Bra. bromeliae* SPC951	71.5	74.0	32.7	65.8	Yes*	*Nos.* sp. FACHB-152	29.0	31.0	35.1		No	
*Bra. sennae* CENA114	69.9	74.0	34.7	65.3	No*	*Nos.* sp. FACHB-280	28.9	34.5	33.2		No	
*Bra.* sp. CT11	67.9	73.4	33.1	65.0	Yes*	*Nos.* sp. FACHB-892	28.2	32.9	34.3	26.2	No	
*Bra.* sp. UFV-L1	71.4	80.0	71.9	63.7	**Yes**	*Nos.* sp. FACHB-973	29.0	33.2	38.0		No	
*Cal. anomala* FACHB-343	27.6	33.3	33.9		No	*Nos.* sp. KVJ20	79.5	81.4	77.4	69.7	**Yes**	
*Cal. brevissima* NIES-22	28.1	33.3	34.0		No	*Nos.* sp. LEGE 06077	29.1	32.7	37.0		No	
*Cal. desertica* PCC 7102	69.8	70.4	35.7	67.7	Yes*	*Nos.* sp. LEGE 12450	27.2	32.1	37.8		No	
*Cal. elsteri* CCALA 953	71.2	69.7	34.7	68.5	Yes*	*Nos.* sp. MG11	29.0	33.3	33.0		No	
*Cal. membranacea* FACHB-236	27.9	33.3	32.6	23.2	No	*Nos.* sp. MS1	28.5	32.8	28.6		No	
*Cal. parasitica* NIES-267	61.8	70.4	34.4	62.0	No	*Nos.* sp. N6	28.3	32.5	34.3		No	
*Cal. rhizosoleniae* SC01	62.7	71.4	64.7	60.3	No*	*Nos.* sp. NIES-3756	27.9	33.4	33.2	27.3	No	
*Cal.* sp. 336/3	27.7	33.8	36.0		No	*Nos.* sp. NIES-4103	27.5	32.3	34.3		No	
*Cal.* sp. HK-06	28.2	69.1	36.5	66.5	No	*Nos.* sp. PA-18–2419	71.0	81.5	72.8	65.3	No*	
*Cal.* sp. NIES-2098	28.3	32.2	34.8		No	*Nos.* sp. PCC 7107	28.1	33.0	33.9		No	
*Cal.* sp. NIES-2100	28.4	34.5	38.6		No	*Nos.* sp. PCC 7120	81.0	82.9	76.6	70.1	**Yes**	
*Cal.* sp. NIES-3974	73.4	68.1	35.1	63.4	No	*Nos.* sp. PCC 7524	76.5	82.6	71.6	70.0	**Yes**	
*Cal.* sp. NIES-4105	69.1	69.9	36.7	66.8	Yes*	*Nos.* sp. T09	28.6	33.3	36.8		No	
*Cal.* sp. PCC 6303	70.6	68.7	33.1	67.6	Yes*	*Nos.* sp. TCL240-02	71.1	86.8	77.9	71.4	**Yes**	
*Cal.* sp. PCC 7103	70.8	68.2	35.7	67.3	Yes*	*Nos.* sp. TCL26-01	28.2	34.4	33.7		No	
*Cal.* sp. PCC 7507	75.5	80.7	77.7	71.7	**Yes**	*Nos.* sp. UCD121	26.9	32.4	34.7	25.0	No	
*Cal.* sp. PCC 7716	68.7	70.4	35.7	67.3	No	*Nos.* sp. UHCC 0702	27.5	32.0	38.1		No	
*Chl. fritschii* PCC 6912	28.0	29.6	33.1	23.1	No	*Nos.* sp. UHCC 0870	76.7	82.3	74.6	68.6	No	
*Cus. issatschenkoi* CHARLIE-1	28.3		30.9		No	*Nos.* sp. WHI	27.6	32.1	36.2		No	
*Cyl. curvispora* GIHE-G1	28.1		30.5		No	*Nos.* sp. XA010	28.3	33.1	36.1		No	
*Cyl. raciborskii* Cr2010	27.9		30.5		No	*Nos. sphaeroides* Kutzing	27.7	32.8	37.8		No	
*Cyl.* sp. FACHB-282	28.8	33.8	38.1		No	*Nos. spongiaeforme* FACHB-130	29.4	33.3	34.3		No	
*Cyl. stagnale* PCC 7417	28.0	33.5	38.2		No	*Ple.* cf*. radiosum* LEGE 06105	59.5	70.4	33.1	61.7	Yes*	
*Den. phyllosphericum* CENA369	67.8	81.8	73.1	69.4	**Yes**	*Ric. intracellularis* HH01			28.5		No	
*Des. muscorum* FACHB-395	71.1	86.7	76.5	70.7	**Yes**	*Ric. sinica* FACHB-800	75.9	81.2	70.9	70.3	**Yes**	
*Dol. circinale* AWQC310F	29.4		30.2		No	*Riv.* sp. PCC 7116	29.0	67.3	35.7	65.2	No*	
*Dol. compactum* NIES-806	92.1	87.6	85.1	85.9	**Yes**	*Roh.* sp. LEGE 12411	28.8	34.1	34.0		No	
*Dol. flos-aquae* CCAP 1403/13F	92.0	87.1	86.1	85.5	**Yes**	*Scy. hofmanni* UTEX2349	30.0	34.8	33.6		No	
*Dol. planctonicum* NIES-80	28.5		30.2		No	*Scy. hofmannii* PCC 7110	28.6	80.6	71.9	61.9	No	
*Dol.* sp. FACHB-1091	98.9	98.9	99.1	98.5	**Yes**	*Scy.* sp. HK-05	28.3	34.8	37.0		No	
*Dol.* sp. LEGE 00240	91.1	87.6	87.5	86.4	**Yes**	*Scy.* sp. NIES-4073	27.9	34.3	33.5		No	
*Dol.* sp. UHCC 0259	28.4	32.0	33.5		No	*Scy.* sp*. UIC* 10036	68.7	80.6	72.8	60.7	**Yes**	
*Fis. muscicola* PCC 7414	28.6	30.2	33.9		No	*Scy. tolypothrichoides* VB-61278	28.8	73.2	35.4	64.7	No	
*Fis. muscicola* SAG 1427–1	28.1	31.8	35.8		No	*Sph. aphanizomenoides* BCCUSP55	28.1	32.5	36.3		No	
*Fis.* sp. PCC 9431	29.1	30.1	33.3		No	*Sph. kisseleviana* NIES-73	77.6	81.6	74.6	66.7	**Yes**	
*Fis.* sp. PCC 9605	29.5	30.6	34.7		No	*Sph. torques-reginae* ITEP-024	77.2	80.5	74.4	67.7	**Yes**	
*Fis. thermalis* PCC 7521	69.0	75.2	69.0	64.5	**Yes**	*Tol. bouteillei* VB521301	28.0	79.6	71.6	61.1	No	
*For. contorta* PCC 7126	74.3	78.6	73.4	66.3	**Yes**	*Tol.* sp. FACHB-123	27.5	33.1	33.7		No	
*For.* sp. LEGE XX443	28.6	32.8	33.5		No	*Tol.* sp. NIES-4075	30.6	33.2	33.5		No	
*Has. byssoidea* VB512170	67.5	71.7	32.6	63.1	Yes*	*Tol.* sp. PCC 7910	28.1	31.6	37.5		No	
*Ini. tapete* BLCC-T55	71.5	71.3	71.0	65.9	No	*Tol. tenuis* PCC 7101	28.7	33.1	37.3		No	
*Kom.* sp. 'clone 1'	78.5	82.4	76.6	70.5	**Yes**	*Tri.* sp. NMC-1	90.4	88.7	85.8	87.6	**Yes**	
*Mas. repens* PCC 10914	28.4	29.6	35.1		No	*Tri. variabilis* 0441	75.2	80.8	71.2	69.8	**Yes**	
*Mas. testarum* BC008	62.4	66.8	35.2	63.4	No*	*Wes. prolifica* IICB1	25.3	30.8	35.8		No	

The corresponding full genus names are as follows: Aet. = Aetokthonos; Ama. = Amazonocrinis; Ana. = Anabaena; Aph. = Aphanizomenon; Atl. = Atlanticothrix; Aul. = Aulosira; Bra. = Brasilonema; Cal. = Calothrix; Chl. = Chlorogloeopsis; Cus. = Cuspidothrix; Cyl. = Cylindrospermopsis; Den. = Dendronalium; Des. = Desmonostoc; Dol. = Dolichospermum; Fis. = Fischerella; For. = Fortiea; Has. = Hassallia; Ini. = Iningainema; Kom. = Komarekiella; Mas. = Mastigocladopsis; Nod. = Nodularia; Nos. = Nostoc; Ple. = Plectonema; Ric. = Richelia; Riv. = Rivularia; Roh. = Roholtiella; Scy. = Scytonema; Sph. = Sphaerospermopsis; Tol. = Tolypothrix; Tri. = Trichormus; Wes. = Westellopsis.

Only one *Nostocales* strain, *Dol.* sp. LEGE 00240, was identified as containing a putative SXT-associated biosynthetic cluster (Table 2, Table S4a and b). The operon spans 25 kb and shares high similarity in gene arrangement and nucleotide alignment (94.9%) with *Dolichospermum circinale* AWQC131C and *Aph. gracile* NH-5 [77, 78]. The *sxt* gene cluster encodes key enzymes involved in SXT biosynthesis, including *sxtA*, which initiates the formation of the toxin’s tricyclic structure, and *sxtE*, which is essential for structural modifications [77, 79]. Variations in the *sxt* cluster across cyanobacterial species contribute to the production of SXT analogues such as neoSXT and gonyautoxins (Fig. 3) [69, 80]. While SXT biosynthesis in *Dol.* sp. LEGE 00240 requires experimental verification, this is the first report suggesting its potential to harbour an SXT-associated gene cluster.

Our analysis identified seven *Nostocales* strains with putative MYC- or NOD-associated biosynthetic clusters: *Iningainema tapete* BLCC-T55, *Nodularia spumigena* CCY9414, *Nos.* sp. CENA543, *Nos.* sp. CHAB 5836, *Nos.* sp. CMAA1605, *Nos.* sp. WHI and *Westelliopsis prolifica* IICB1 (Table 2, Fig. 3, Tables S5a and b). These genes were identified using *mcy* sequences from *Mic. aeruginosa* PCC 7806, as *mcy* and *nda* genes show substantial sequence and structural similarity [81]. *Nos.* sp. CENA543 and *Nod. spumigena* CCY9414 are well-characterized NOD producers, while the remaining five strains have not been previously associated with MYC or NOD biosynthetic clusters [82, 83]. Operon arrangements suggest that *Nos.* sp. CHAB 5836, *Nos.* sp. WHI and *Wes. prolifica* IICB1 contain MYC-like clusters, with operons including core *mcy* genes such as *mcyA*, *mcyB* and *mcyC*, which are involved in assembly of the MYC heptapeptide structure [84, 85]. Conversely, *Ini. tapete* BLCC*-*T55 and *Nos.* sp. CMAA1605 exhibited features more consistent with NOD-like clusters, including *ndaA* and *ndaB*, which encode core nonribosomal peptide synthetase/polyketide synthase components involved in NOD biosynthesis [81, 84]. Notably, in *Nos.* sp. CMAA1605, the *nda* cluster is fragmented, with truncated genes (e.g. *ndaC* is ~3 kb compared to ~7 kb in *Nos.* sp. CENA543) likely reflecting incomplete NOD synthesis capability or a genome assembly artefact due to the genome’s 90.8% completeness [83]. Experimental validation is required to confirm toxin biosynthesis in these isolates [86].

In agreement with previous literature, strains of *Anabaena*, *Dolichospermum*, *Cuspidothrix* and *Cylindrospermopsis* were among the main genera with ATX- and SXT-associated BGCs [2, 12, 87]. MYC- and NOD-associated genes were detected mainly in *Nostoc* and *Nodularia* strains, consistent with previous reports [2]. These findings are also consistent with Österholm *et al*. [14], who showed that cyanotoxin and secondary metabolite BGCs within the *ADA* clade are unevenly distributed and tend to follow phylogenomic subgrouping rather than genus labels. However, unlike the Österholm *et al*. dataset, in which MYC/NOD-associated clusters were identified in some *Anabaena* and *Dolichospermum* strains, none of the *Anabaena* or *Dolichospermum* genomes included in the present analysis contained coherent MYC/NOD-associated BGCs [12, 14]. This discrepancy most likely reflects differences in genome sampling rather than a true absence of MYC/NOD potential from these genera. The present study also highlights putative MYC/NOD-associated BGCs in the *Westelliopsis* and *Iningainema* genera, supporting previous evidence that cyanotoxin-associated biosynthetic potential extends beyond the most commonly reported bloom-forming genera [88].

Finally, CYR biosynthetic genes were not identified in any of the *Nostocales* genomes in this study (Table S6a and b). It has previously been reported that CYR biosynthesis is highly strain-specific even within genera that include known CYR producers, such as *Cylindrospermopsis* and *Dolichospermum* [89]. Consistent with this interpretation, the presence of *Cylindrospermopsis*- or *Dolichospermum*-labelled genomes in the present dataset does not imply that these particular strains are expected to carry CYR-associated clusters. Rather, the absence of CYR BGCs most likely reflects the specific strain composition of the dataset, in which the available *Cylindrospermopsis* and *Dolichospermum* genomes did not correspond to confirmed CYR-producing lineages. Nevertheless, because the screen was reference-based, the presence of highly divergent, fragmented or poorly assembled CYR-associated clusters cannot be fully excluded. Broader sampling of confirmed CYR-producing *Nostocales* genomes will therefore be needed to assess the distribution of CYR biosynthetic potential more comprehensively.

Overall, out of the 164 genomes analysed, 12 strains (7.3%) were found to harbour putative cyanotoxin-associated BGCs, primarily associated with ATX, MYC and NOD. Importantly, none of the *bona fide AFA* strains discussed in section 3.2 possessed any of these gene clusters. This suggests that cyanotoxin-associated biosynthetic potential is restricted to a limited fraction of the analysed *Nostocales* strains, highlighting the importance of accurate genomic screening in lakes and blooms containing multiple co-occurring cyanobacteria and microalgae. In this context, Dreher *et al*. [12] demonstrated the value of metagenomic sequencing of HAB samples for identifying cyanotoxin biosynthetic operons within associated cyanobacterial genomes [12]. Clearly, the presence of cyanotoxin-associated biosynthetic genes in these genomes indicates potential toxin biosynthesis, which requires experimental confirmation [71, 74]. This is particularly important where candidate BGCs differ from known reference organisms, as observed with *Wes. prolifica* IICB1 and *Dolichospermum* LEGE 00240 (Fig. 3). To validate toxin production and determine the exact nature of the metabolites produced, targeted assays such as ELISA or liquid chromatography-mass spectrometry (LC-MS) should be used, particularly to assess whether these clusters produce known toxins, less potent analogues or distinct secondary metabolites [80].

### Most *Nostocales* species, including *AFA*, possess genes associated with natural competence

Natural competence enables bacteria to take up extracellular DNA and contributes to horizontal gene transfer, genetic diversity, adaptation and genome evolution [90]. In cyanobacteria, this process is commonly associated with type IV pili, which bind extracellular dsDNA and contribute to its transport across the cell envelope. During uptake, DNA is processed into ssDNA, which is then protected by competence-associated proteins and loaded onto RecA to facilitate homologous recombination into the host genome [91]. Key *pil* genes including *pilA*, *pilB*, *pilC*, *pilD*, *pilM*, *pilN*, *pilO*, *pilP* and *pilQ* encode proteins involved in pilus assembly, extension, retraction and surface-associated DNA binding, while *comEA*, *comEC* and *comF* are associated with DNA binding and translocation into the cytosol [92]. Additional genes, including *dprA*, *hfq* and *ebsA*, may contribute to DNA protection, regulation or recombination during the competence process. Together, these genes provide a genomic basis for assessing natural competence potential amongst the *Nostocales* [91, 93].

**Fig. 4.**
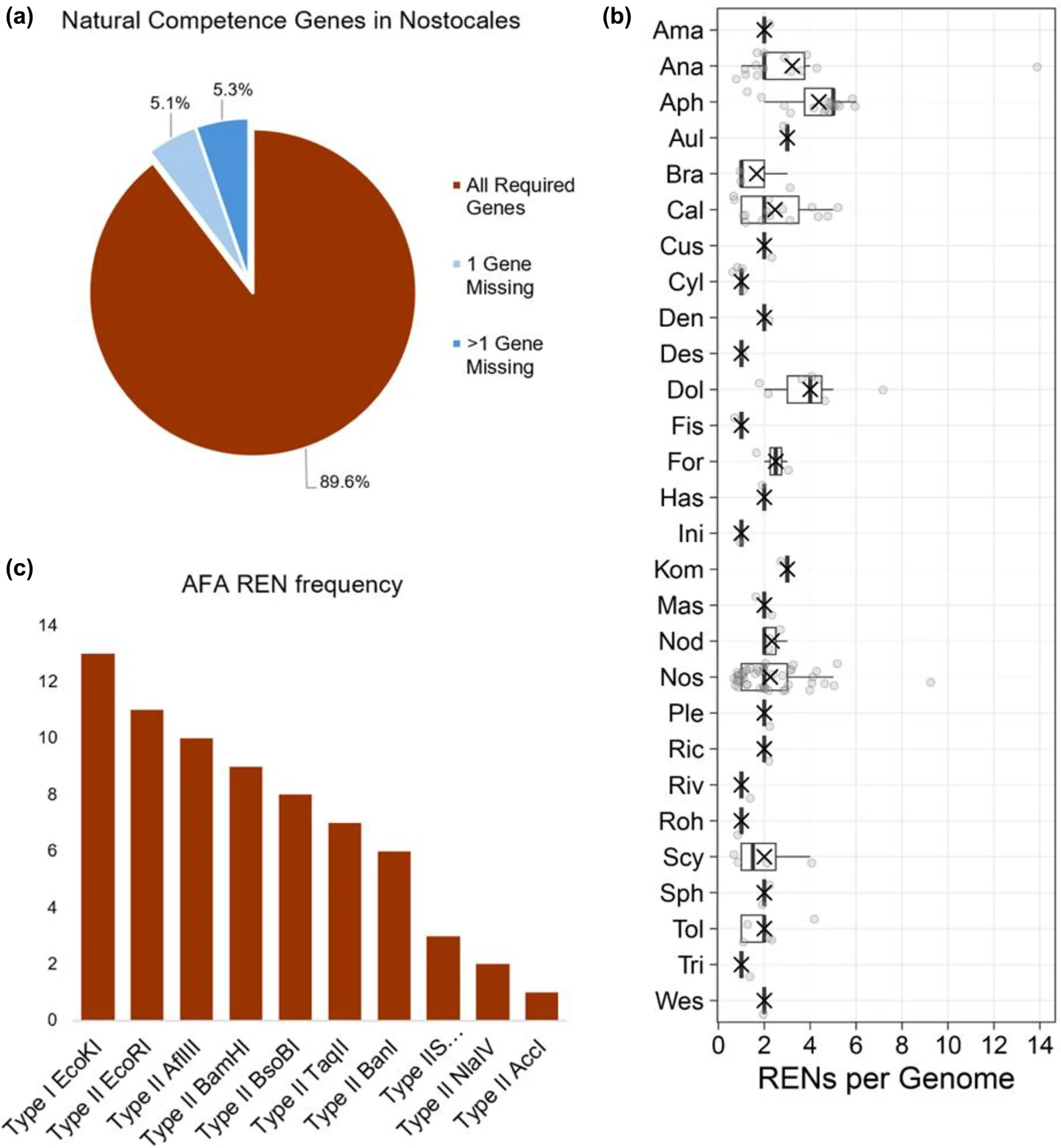
Natural competence gene presence and putative R–M system-associated proteins within the analysed *Nostocales* and *AFA* genomes. (**a**) Pie chart illustrating the percentage of genomes containing the full set of analysed natural competence-associated genes, missing one gene or missing more than one gene. (**b**) Boxplot showing putative R–M system-associated proteins per genome across *Nostocales* genera. Grey points represent individual genomes. Mean: cross; median: solid line. (**c**) Bar chart representing the frequency of different putative R–M system-associated proteins in *AFA* genomes.

Using blastp searches of the 164 genomes, we found that 147 genomes contain the full set of natural competence-associated genes (Table S8a and b), indicating that the majority (89.6%) of the genomes possess the genetic potential for natural competence [90] (Fig. 4a). While recent studies have demonstrated the natural transformation of two species, *Phormidium lacuna* and *Chlorogloeopsis fritschii* PCC 6912 [26, 94], transformation of filamentous cyanobacteria such as *Anabaena* sp. and *Aphanizomenon* sp*.* is complicated by having to transform whole filaments, which includes intact extracellular polysaccharide layers that impede DNA access, DNA degradation by host restriction systems and poorly defined strain-specific barriers to DNA uptake [95, 96]. Therefore, while most analysed *Nostocales* genomes possess the genetic potential for natural competence, experimental optimization is required to determine which strains can be genetically transformed through uptake of exogenous DNA.

Currently, the most widely used genetic engineering technique for *Nostocales* is conjugation, which enables transfer of plasmid DNA from a donor bacterium, typically *Escherichia coli*, to the cyanobacterial recipient through direct cell-to-cell contact [31, 95]. Successful conjugation in the laboratory requires a ‘cargo’ plasmid, which carries the gene of interest, and a ‘conjugative’ plasmid, which allows the *E. coli* to transfer the cargo plasmid to the recipient cell [97, 98]. Compared to natural transformation, conjugation involves a self-replicating plasmid being delivered into the recipient cell, instead of ssDNA that requires incorporation into the genome. However, incoming plasmid DNA can still be targeted by host R–M systems, which can degrade unmethylated or incorrectly methylated DNA [99, 100].

Restriction of incoming plasmid DNA by host R–M systems is well established in *Anabaena* sp. PCC 7120. Elhai and Wolk developed a specific conjugation methodology to protect target DNA from the host’s restriction endonuclease isoschizomers of *Ava*I, *Ava*II and *Ava*III [99]. Their strategy to overcome this was to use a third ‘helper’ plasmid that methylated the specific DNA sequences within the cargo plasmid, which would otherwise be targeted by the recipient *Ava* RENs [30]. The use of this additional plasmid has enabled the routine generation of *Anabaena* sp. PCC 7120 transformants [31]. In light of the importance of R–M systems to transformation, we investigated our genomic dataset for genes encoding R–M system-associated proteins that may compromise the acquisition of conjugative cargo plasmids.

We identified that the *AFA* genomes had the highest number of putative R–M system-associated proteins per genome, with an average of four or more per genome and a median of five (Fig. 4b). *AFA* was followed closely by *Dolichospermum* strains, with an average of nearly four, and *Anabaena* strains, with an average of ~three. Fig. 4c further details the different types of putative R–M system-associated proteins detected within *AFA* genomes. Similar restriction-related systems have previously been reported in *Ana*. sp. 90 [101]. The complete list of analysed R–M system-associated proteins is provided in Table S9a. To strengthen these annotation-based predictions, all 15 putative R–M system-associated proteins identified in the three newly sequenced *AFA* genomes were cross-validated against REBASE. All had REBASE matches consistent with R–M system-associated functions, including Type I methyltransferase or specificity subunits, Type II restriction enzymes and Type IIG R–M enzymes (Table S9b).

Due to the widespread distribution of natural competence-associated genes across the analysed *Nostocales* genomes, the presence of R–M system-associated proteins may represent an important barrier modulating horizontal gene transfer, especially in species such as *AFA* [102]. The natural competence gene profiles and R–M system-associated protein data presented here may therefore support the design of tailored transformation protocols for specific *Nostocales*, including selection of target strains, methylation-compatible DNA preparation and strategies to bypass host restriction barriers. However, some R–M systems may have been missed owing to annotation limitations or the presence of novel, uncharacterized systems, while some annotated R–M system-associated proteins may not have the predicted function or recognition specificity. Experimental validation is therefore required to determine how these systems affect transformation efficiency in individual strains.

### Klamath *AFA* may acquire vitamin B_12_ from commensal bacteria

**Fig. 5.**
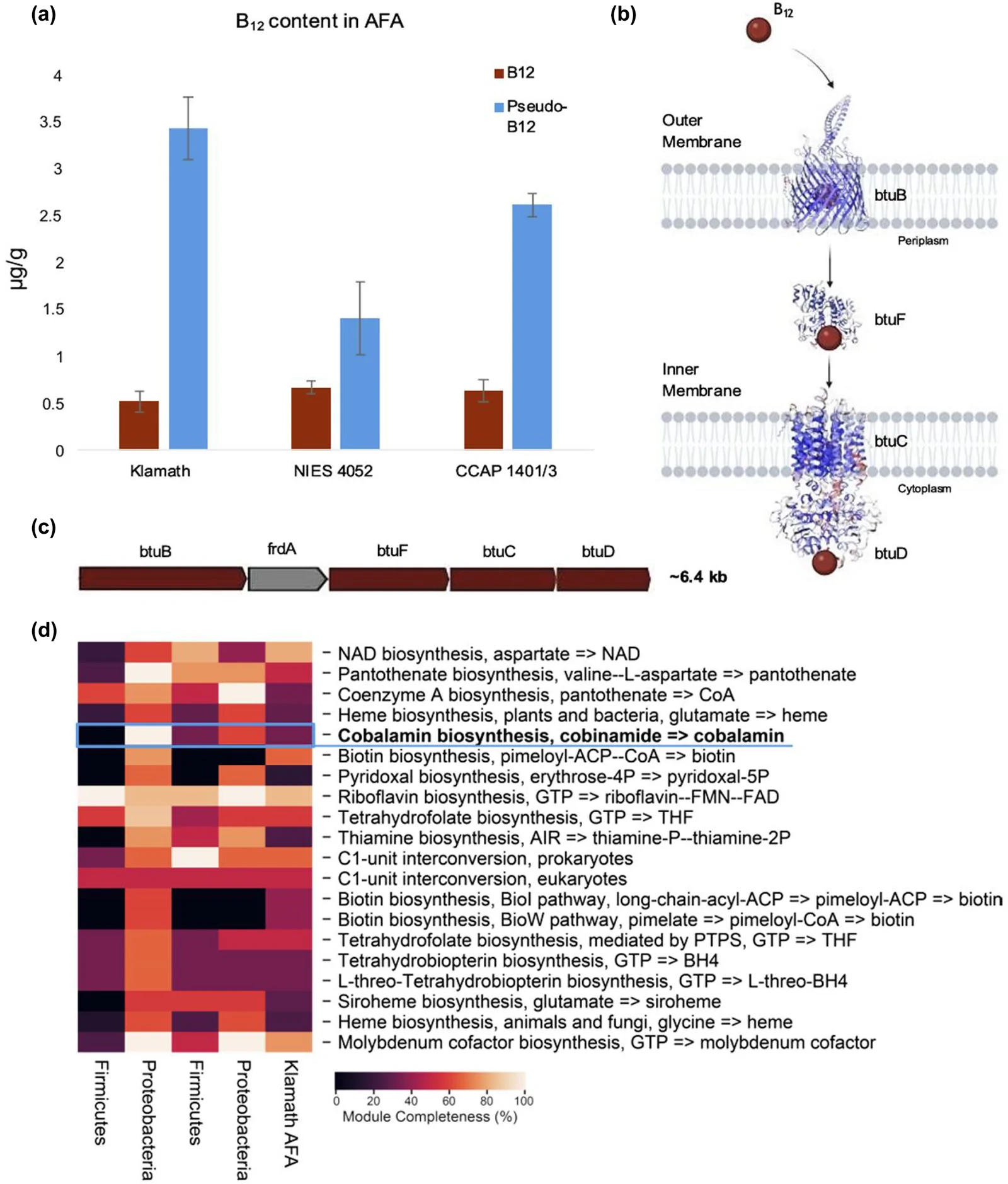
B_12_ and pseudo-B_12_ levels in *AFA*, the *btu* B_12_ import system and *AFA*’s commensal B_12_-producing bacteria. (**a**) Bar chart highlighting the content of B_12_ and pseudo-B_12_ in Klamath *AFA*, NIES 4052 *AFA* and CCAP 1401/3 *AFA*. (**b**) Illustration of the cobalamin import pathway composed of btuB, btuF, btuC and btuD. (**c**) *btu* operon in Klamath *AFA*. (**d**) Metabolic summary heatmap of cofactors and vitamins in Klamath *AFA* and its four most abundant commensal bacteria. The four bacterial columns correspond to four distinct metagenomic bins [metagenome-assembled genomes (MAGs)], comprising two *Firmicutes* MAGs and two *Proteobacteria* MAGs, which represent different bacterial taxa identified in the sample. Specifically, the two *Firmicutes* MAGs were classified at the genus level as *Lactococcus* (order *Lactobacillales*) and *Clostridium* (order *Clostridiales*), while the two *Proteobacteria* MAGs were classified as *Pseudomonas* (order *Pseudomonadales*) and *Limnohabitans* (order *Burkholderiales*). The WGS metabolic heatmap summary for both Klamath and CCAP 1401/3 *AFA* can be viewed in Figs S2 and S3. The blue line highlights the cobalamin pathway. The colour indicates module completeness – i.e. the percentage of genes present required to produce the cofactor/vitamin in question, with white as 100% and black as 0%.

An ongoing question is whether Klamath *AFA*-associated biomass contains nutritionally relevant vitamin B_12_ (= cobalamin), and whether reported levels arise from associated commensal bacteria rather than synthesis by the cyanobacterium itself [103]. Cyanobacteria are generally considered to synthesize pseudo-B_12_ rather than true B_12_ [32, 104]. Compared to B_12_, pseudo-B_12_ has a 500-fold lower binding efficiency to human intrinsic factor – the transporter in humans that allows for B_12_ absorption in the ileum [105]. Structurally, the difference between the two B_12_ forms is minimal and relates to the lower axial ligand binding the cobalt ion: in B_12_ the ligand is 5,6-dimethylbenzimidazole (DMB), while in pseudo-B_12_, DMB is replaced by adenine [9]. Critically, cyanobacteria, including *AFA*, lack the genes required for DMB biosynthesis and are therefore expected to produce pseudo-B_12_ rather than true B_12_ [32, 106]. Consistent with this, we found that *AFA* strains contained pseudo-B_12_ as the dominant corrinoid form (Fig. 5a).

Pseudo-B_12_ abundance varied between cultures, most likely reflecting differences in pseudo-B_12_ production, culture density and growth state. In contrast, true B_12_ was detected at ~0.5 µg g^−1^ biomass across the three cultures. An earlier spectroscopy study also obtained the same B_12_ concentration for a Klamath *AFA* [33]. To investigate the likely source of B_12_, we assessed cobalamin biosynthesis pathway completeness in the commensal bacteria metagenome-assembled genomes (MAGs) associated with the *AFA* cultures using Illumina metagenomic sequencing and MicrobeAnnotator [50]. In all three *AFA* cultures, bacterial MAGs with predicted B_12_ biosynthesis potential were identified (Fig. 5d). In the Klamath *AFA* metagenome, the strongest candidate was a gammaproteobacterial MAG classified within *Pseudomonas*. This MAG was 96.02% complete, 3.41% contamination and had a relative abundance (based on mapping DNA reads to the assembled MAG) of 16.3%. This was of a comparable relative abundance to the corresponding Klamath *AFA* MAG at 15.7% relative abundance. MicrobeAnnotator predicted a complete cobalamin biosynthesis pathway in this *Pseudomonas* MAG, supporting it as the most likely bacterial contributor to the measured B_12_ pool. Although this remains a pathway-level bioinformatic prediction rather than direct experimental evidence of B_12_ production, these findings support the hypothesis that associated heterotrophic bacteria contribute to the B_12_ detected within non-axenic *AFA* biomass.

We further investigated whether vitamin B_12_ was actively transported into the cytosol of *AFA*. The *Nostocales* genomes present a clear need for B_12_ as all 164 possess *metH*, a gene encoding a methionine synthase which requires cobalamin as a cofactor and which has a preference for B_12_ over pseudo-B_12_ [32]. Only 11 genomes were found to also contain the *metE* gene, which encodes an alternative, B_12_-independent form of methionine synthase (Table S10a and b). Furthermore, a B_12_ import system, btu (*btuB*, *btuC*, *btuD* and *btuF*), has been described for cyanobacteria [35] and experimentally validated in *Synechococcus* sp. PCC 7002 [107] (Fig. 5b). The *btu* genes encode proteins that work together to facilitate the transport of B_12_ across the bacterial membrane [35]. BtuB encodes an outer membrane receptor that binds B_12_, whereas BtuC and BtuD form a transmembrane complex responsible for transporting B_12_ across the inner membrane, driven by ATP hydrolysis [108]. BtuF functions as a periplasmic binding protein that shuttles B_12_ to the transport complex (BtuCD) [109, 110]. We investigated what percentage of *Nostocales* genomes contained these four *btu* genes and if they were arranged within an operon as found in *Synechococcus* sp. PCC 7002.

Out of the 164 genomes analysed, 42 genomes (25.6%) had all four *btu* genes, 18 (11.0%) had three of the *btu* genes, and four (2.4%) had two. Notably, 38 (23.2%) of the 42 genomes possessed the same *btu* operon layout, *btuB–frdA–btuF–btuC–btuD* (Table 3), as originally described in *Anabaena variabilis* ATCC 29413 [35]. This shared arrangement was detected across multiple *Nostocales* genera in the present dataset, suggesting a conserved *btu* operon configuration potentially characteristic of *Nostocales* strains. Furthermore, 10 (6.4%) of the 18 genomes containing three *btu* genes showed the same operon arrangement but lacked *btuD* (Table 3). A further six (3.6%) genomes had operons containing two or more *btu* genes, but with a completely different arrangement (Table 3). In summary, 64 genomes (39%) were found to contain two or more *btu* genes, with 54 (32.9%) of these arranged in a putative operon. Among these, 48 genomes (29.2%) exhibited the shared *btuB–frdA–btuF–btuC–btuD* arrangement or the same arrangement lacking *btuD*. Overall, a substantial proportion of the analysed *Nostocales* strains, including Klamath *AFA*, possess the potential to import B_12_ through a *btu*-dependent transport system. Further comparative analysis across a broader cyanobacterial dataset would be required to determine whether this operon configuration is specific to *Nostocales* or more widely distributed among cyanobacteria.

Previous studies have highlighted the redundancy of *btu* genes, especially the gene encoding the ABC transporter, BtuD. For example, the *btu* operon in *Synechococcus* sp. PCC 7002 was shown to be a functional B_12_ import system, but *btuD* was not found in the genome [35]. Similarly, our analysis shows that out of all the genomes missing one or more *btu* genes (*n*=22), *btuD* was missing in 19. This may reflect limited detection of divergent *btuD* homologues by sequence-searching and annotation approaches or functional sharing of ATPase subunits among ABC transporters in cyanobacteria [35]. Among genomes missing one or more *btu* genes, *btuB* was absent from five genomes and *btuC* from one genome. As these components are more specific to cobalamin transport, redundancy is less likely in these cases, although it cannot be excluded. In contrast, *btuF* was detected in all 64 genomes containing at least one *btu* gene, making it the most consistently detected *btu* marker in this dataset. Overall, while not all 64 genomes possess the shared *btuB-frdA-btuF-btuC-btuD* arrangement, the presence of multiple *btu* components suggests that many might still encode a functional or partially divergent B_12_ import system.

### Experimental validation of B_12_

**Fig. 6.**
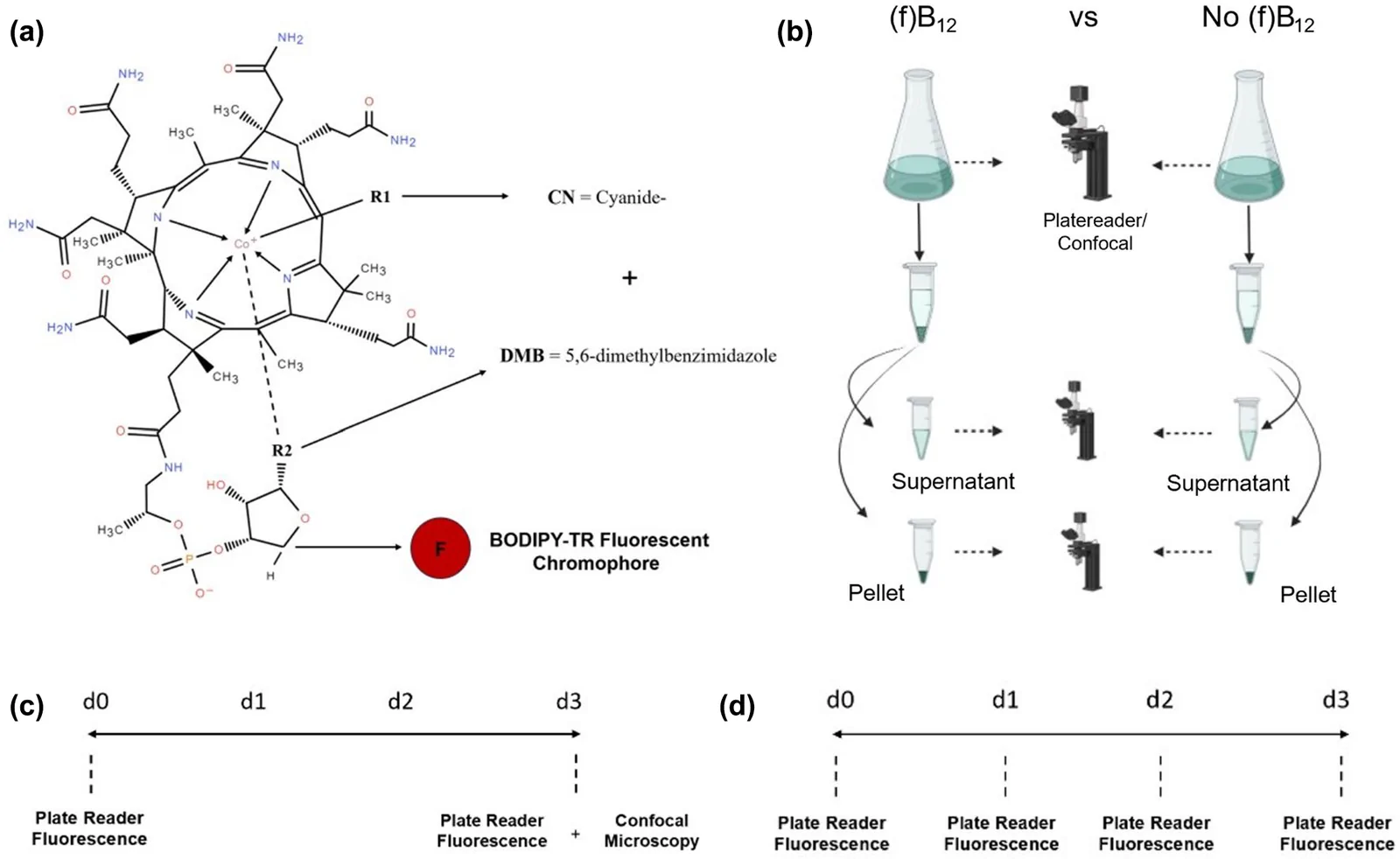
Methodology used to assess fluorescent B_12_ analogue uptake in *AFA* strains. (**a**) Visual representation of the fluorescent B_12_ (cyanocobalamin) analogue, BODIPY-TR-B_12_ [(f)B_12_], which has the fluorescent chromophore BODIPY-TR, attached to the ribose moiety of the B_12_ nucleotide loop. The excitation/emission maxima of BODIPY-TR-B_12_ are 588/621 nm. (**b**) Illustration of the experimental methodology employed: *AFA* cultures were grown in the presence and absence of (f)B_12_ for up to 3 days and the harvested pellet and supernatant were tested for (f)B_12_ fluorescence (584/620 nm). (**c**) Experimental timeline for Fig. 7a and b, in which pellet-associated (f)B_12_ fluorescence (584/620 nm) was measured on day 3 using a plate reader and confocal microscopy. (**d**) Experimental timeline for Fig. 7d, e, f and g, in which pellet-associated (f)B_12_ fluorescence was measured on days 0, 1, 2 and 3 using plate-reader analysis.

**Fig. 7.**
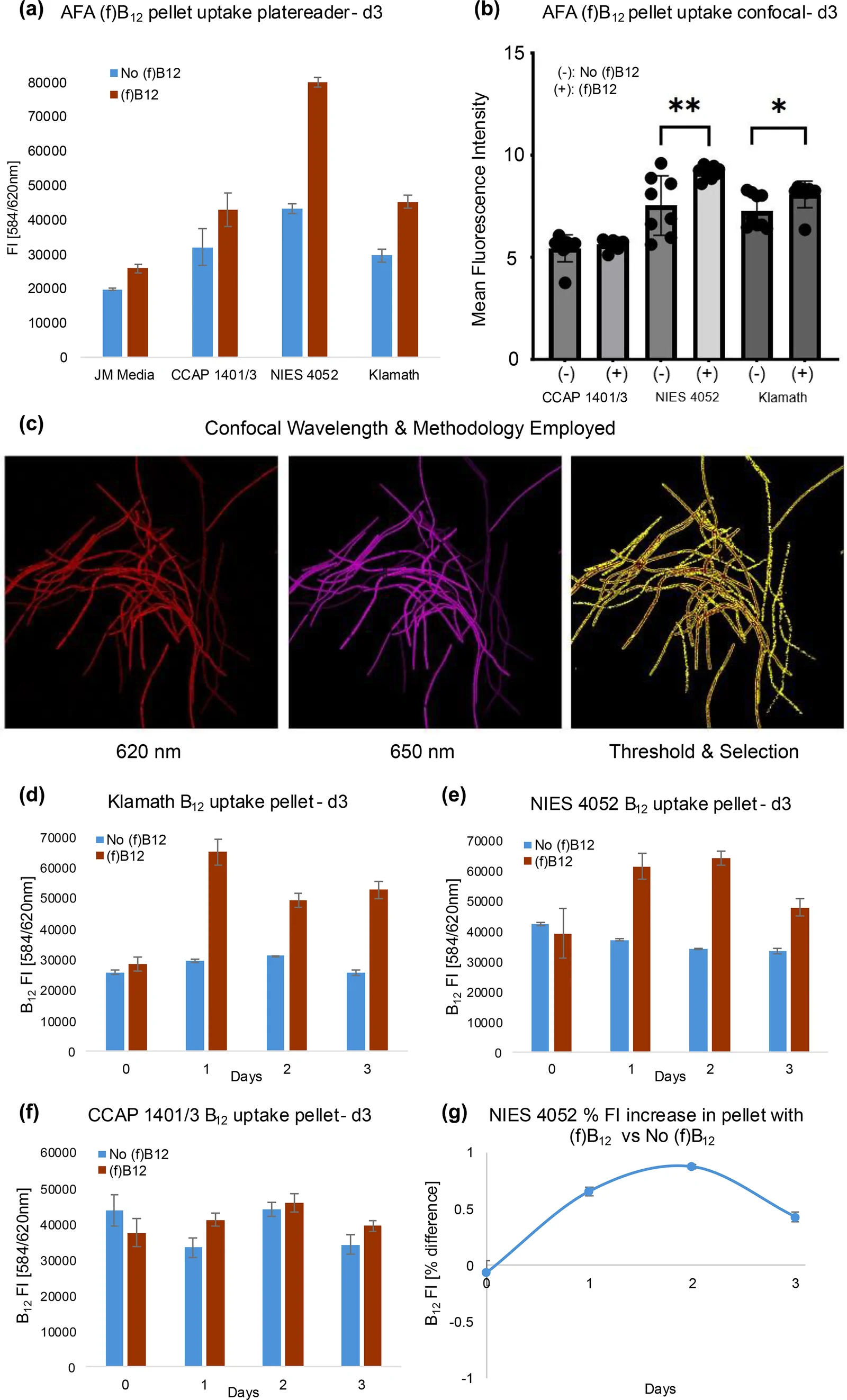
*AFA* (f)B_12_ uptake within cells. (**a**) and (b) both show the (f)B_12_ fluorescence ratio [584/620 nm] measured in the pellet of CCAP 1401/3, NIES 4052 and Klamath *AFA* incubated for 3 days in JM medium with or without (f)B_12_. (**c**) Representative confocal images showing the image-analysis workflow used to quantify *AFA*-associated fluorescence. The 620 nm channel corresponds to the (f)B_12_/BODIPY-TR signal, while the 650 nm channel corresponds to endogenous phycocyanin fluorescence. The 650 nm channel was used to generate a thresholded *AFA* filament mask, shown in the right-hand image, which was then applied to the 620 nm channel to quantify *AFA*-associated (f)B_12_ fluorescence. This thresholding approach restricted analysis to *AFA* filament-associated signal and reduced the contribution of free or weakly associated bacterial cells. (**d**), **(e**) and (f) bar charts showing (f)B_12_ uptake within cells by comparing pellet fluorescence over 3 days, while (g) indicates the % (f)B_12_ fluorescence increase in NIES 4052 *AFA* pellets grown with or without (f)B_12_ over the same period. *, significant (*P*≤0.05) Student’s t-test. Error bars show sd of triplicate values.

We experimentally validated B_12_ uptake in CCAP 1401/3, NIES 4052 and Klamath *AFA* using the fluorescent analogue (f)B_12_ (Fig. 6a). Two experiments were performed to measure (f)B_12_ fluorescence in the culture supernatant and cell pellet (Fig. 6b). In the first experiment, fluorescence analysis was performed at days 0 and 3 using a plate reader and confocal microscopy (Fig. 6c). In the second experiment, measurements were taken repeatedly over 3 days using only the plate reader (Fig. 6d). To assess (f)B_12_ uptake, we examined B_12_ uptake in the cell pellet by fluorescence measurement relative to the background fluorescence of untreated cells to account for fluorescence arising from endogenous pigments (Fig. 7c).

For the first experiment, we found that on day 3 the cell pellet of NIES 4052 *AFA* and Klamath *AFA* grown with (f)B_12_ had a significantly higher 620 nm fluorescence than the negative control, with NIES 4052 *AFA* having approximately double the fluorescence (Fig. 7a). For CCAP 1401/3 *AFA,* the difference was not significant (Fig. 7a). This result was corroborated by confocal microscopy, with Klamath and NIES 4052 *AFA* having a higher (f)B_12_ pellet fluorescence than the control (Fig. 7b). A second experiment was performed to confirm the previous findings and track the uptake of (f)B_12_ over the course of 3 days. Fig. 7d and e show that the fluorescence of NIES 4052 and Klamath *AFA* pellets incubated with (f)B_12_ remained the same as that of the control (without (f)B_12_) on day 0 but was significantly higher on days 1, 2 and 3. This suggests that, as before, (f)B_12_ may have entered the cytosol, contributing to the overall cell pellet fluorescence. In contrast, the fluorescence of the CCAP 1401/3 *AFA* pellet remained unchanged throughout the 3 days (Fig. 7f). Finally, Fig. 7g illustrates the increase in 620 nm fluorescence as a percentage over time, peaking on day 2 and decreasing on day 3, which we hypothesize may indicate the export or degradation of the (f)B_12_ analogue. Klamath *AFA* showed a peak on day 1 and remained relatively stable over the next 2 days (Fig. 7d).

From these experiments, 24 h after incubation with (f)B_12_, Klamath and NIES 4052 *AFA* cell pellets exhibited increased 620 nm fluorescence, while CCAP 1401/3 *AFA* did not. Based on the genomic data, Klamath *AFA* is most likely to import the (f)B_12_ via the shared *btuB–frdA–btuF–btuC–btuD* operon arrangement described above [35]. Although NIES 4052 *AFA* showed increased intracellular/cell-associated (f)B_12_ fluorescence, suggesting B_12_ analogue import, it lacks the shared *btuB–frdA–btuF–btuC–btuD* operon arrangement or any other identifiable *btu* operon arrangement. It is important to note that while the NIES 4052 *AFA* genome assembly was highly complete (99.89%), its fragmentation across 80 Illumina-derived contigs may limit detection of the complete operon architecture (Section 3.1). Even so, annotation using Prokka identified a putative *btuB* analogue (NIES_4052_bin-011_02825) and, critically, a *btuF* analogue (NIES_4052_bin-011_02659) (File S1). Furthermore, Prokka identified an additional ten *btuD*-like genes spread throughout the NIES 4052 *AFA* genome, but no putative *btuC* was found (File S1). Therefore, the (f)B_12_ import observed in NIES 4052 *AFA* (Fig. 7) may employ divergent *btu* analogues or a partially unresolved transporter architecture rather than the shared *btuB–frdA–btuF–btuC–btuD* arrangement.

The analysis of CCAP 1401/3 AFA did not indicate uptake of (f)B_12_. Like NIES 4052 *AFA*, it lacks the shared *btu* operon arrangement or any other identifiable *btu* operon, although Prokka annotation identified putative *btuB*, *btuF* and *btuD*-like components. However, the lack of detectable (f)B_12_ uptake may reflect strain-specific transporter specificity, differential B_12_ storage capacity, cobalamin salvage or alternative regulatory mechanisms affecting uptake [34, 111].

To further explore possible regulatory differences, the dataset was screened for cobalamin-related riboswitches using Infernal/Rfam and visualized alongside the *AFA*/*ADA* phylogeny (Fig. S4; Table S12). This analysis revealed a notable phylogenetic pattern: cobalamin riboswitches were detected across much of the broader *Nostocales*/*ADA* dataset, whereas they were largely absent from the core *AFA*-associated group. Specifically, Klamath *AFA* and NIES 4052 *AFA* lacked detectable cobalamin or AdoCbl riboswitches, whereas CCAP 1401/3 *AFA* contained one predicted cobalamin riboswitch but no AdoCbl riboswitch. However, the predicted CCAP 1401/3 cobalamin riboswitch was not located near any putative *btu* gene and therefore cannot currently be linked to canonical *btu*-mediated B_12_ transport regulation. Nevertheless, the observed distribution suggests that *AFA*-associated strains may differ from many related *Nostocales* genomes in their cobalamin-responsive regulatory architecture, which could contribute to strain-specific differences in B_12_ uptake, retention or metabolic response. However, this interpretation remains speculative, and expression-based analysis under varying B_12_ availability would be required to determine whether riboswitch-mediated regulation contributes to the differential (f)B_12_ uptake observed here.

A limitation to our B_12_ uptake study is that BODIPY-TR-B_12_ is a modified cobalamin analogue coupled to the bulky BODIPY-TR fluorophore, and therefore this might alter its affinity for B_12_ transport components relative to native cobalamin. This could contribute to strain-specific differences in fluorescent signal, indicating that the assay should be interpreted as evidence of association with, or uptake of, the fluorescent analogue rather than as a direct quantitative proxy for native intracellular B_12_. This also means that the absence of detectable (f)B_12_ fluorescence cannot be interpreted as definitive absence of native B_12_ uptake, nor can the observed uptake be treated as a direct quantitative reflection of native intracellular B_12_ accumulation. Finally, as all three *AFA* cultures were non-axenic, the bulk B_12_ measurements should be interpreted as B_12_ content of the *AFA*-associated biomass fraction rather than as evidence that all detected B_12_ was intracellularly accumulated by *AFA* cells. As discussed above, the putative cobalamin-producing *Gammaproteobacteria* MAG in the Klamath AFA metagenome represented 16.3% relative abundance, comparable to 15.7% for the corresponding *AFA* MAG, supporting the possibility that associated bacteria contributed to the measured B_12_ pool. As we undertook repeated pellet washing and confocal thresholding based on *AFA* filament fluorescence, the likelihood that the observed fluorescent B_12_ analogue signal was driven primarily by heterotrophic bacterial cells in the biomass pellet is reduced.

Future work should combine direct LC-MS/MS-based cobalamin profiling with defined cyanobacterium–bacteria co-cultures and improved localization assays. In particular, alternative fluorescent B_12_ analogues that emit at wavelengths with lower *AFA* background fluorescence, such as B_12_ Oregon Green, may help resolve intracellular localization more clearly [65]. Further analysis of cobalamin-responsive regulation, *btu* gene expression and uptake under different B_12_ availability conditions would also help determine whether strain-specific regulation contributes to the differential (f)B_12_ uptake observed here.

Overall, we hypothesize that *AFA* strains persist within a cyanobacterial–bacterial consortia, in which associated bacteria contribute true B_12_, while *AFA* may provide other resources such as fixed nitrogen [112–114]. Because true B_12_ is a more suitable cofactor than pseudo-B_12_ for many B_12_-dependent enzymes, this interaction could provide a metabolic advantage to *AFA* strains capable of importing exogenous cobalamin [9, 32]. This interpretation is consistent with the Black Queen Hypothesis, which proposes that organisms may lose or reduce costly metabolic functions when these are supplied by community partners [115]. From a nutritional perspective, the detection of true B_12_ in *AFA*-associated biomass may also help explain previous reports that Klamath *AFA* biomass contains measurable levels of B_12_ [116].

## Conclusions

Our genomic analysis of *AFA* and related *Nostocales* provides important insights into their taxonomy, safety and biotechnological potential. Genome statistics, GTDB-Tk classification and ANI analysis supported the reassessment of several *AFA*-labelled genomes, with CCAP 1401/3, FACHB-1040 and KM1D3_PB separating from the core *AFA* cluster. These results highlight the need for caution when relying on historical NCBI species assignments, particularly when it comes to the ADA clade or filamentous cyanobacteria in general.

In the broader *Nostocales* dataset, this study identifies putative cyanotoxin-associated BGCs in 7.3% of analysed genomes, including six genomes not previously highlighted as potentially carrying cyanotoxin-associated BGCs. The most frequently detected candidate clusters are associated with ATX and MYC/NOD, while the SXT-associated cluster is detected once and CYR-associated clusters are not detected by the present screening framework. These findings should be interpreted as bioinformatic evidence of cyanotoxin-associated biosynthetic potential rather than direct evidence of toxin production. Nevertheless, the combined blastp, operon-structure and antiSMASH-supported analysis provides a useful framework for preliminary safety assessment of *AFA* and related *Nostocales*.

This study also highlights the genetic potential for natural competence in *Nostocales*: the majority of the genomes analysed (89.6%) were found to possess the necessary genes involved in exogenous DNA uptake. However, transformability is not determined by natural competence genes alone, as incoming DNA may also be restricted by strain-specific R–M systems, methylation sensitivity, plasmid compatibility and other host barriers. We therefore analysed all 164 genomes for R–M system-associated proteins to determine which genera might be amenable to genetic transformation. Our results highlight that *AFA, Nostoc, Anabaena* and *Dolichospermum* possessed the highest number of unique R–M system-associated proteins, with *AFA* genomes averaging four per genome. These data may help guide future protocols, including the selection of target strains, methylation-compatible DNA preparation and strategies to bypass host restriction barriers, as observed with the conjugation of *Anabaena* sp. PCC 7002 [30, 99]. However, the R–M analysis remains bioinformatic and will require experimental validation through transformation assays.

Finally, we investigated whether *Nostocales* strains possess the genetic capacity to import B_12_ by searching for *btu* transport genes previously described in cyanobacteria [35]. An identifiable *btu* operon was detected in ~32.9% of the analysed *Nostocales* genomes. The most common arrangement, *btuB–frdA–btuF–btuC–btuD*, was shared across multiple *Nostocales* genera, with variation mainly related to the presence or absence of *btuD*. This suggests a conserved *btu* operon configuration potentially characteristic of *Nostocales* strains, although broader cyanobacterial comparisons would be required to determine its wider distribution.

The B_12_ results support a role for cyanobacterium–bacteria interactions in *AFA*-associated biomass. The three *AFA* cultures analysed were non-axenic and contained associated heterotrophic bacteria with predicted cobalamin biosynthesis potential. This is particularly relevant because cyanobacteria are known to produce pseudo-B_12_ rather than true B_12_. Fluorescent B_12_ analogue uptake experiments showed that Klamath *AFA* and NIES 4052 *AFA* can associate with or import an exogenous B_12_ analogue, supporting a possible route by which bacterially derived B_12_ may become available within *AFA*-associated biomass. These findings may help explain previous reports of measurable true B_12_ in Klamath *AFA* biomass [33, 116]. However, the B_12_ findings should be interpreted with some caution: the B_12_ measurements were obtained from *AFA*-associated biomass, and direct LC-MS/MS-based cobalamin profiling was not performed.

In conclusion, this study provides an integrated genomic and experimental framework for evaluating *AFA* and related *Nostocales*. By combining genome-based taxonomy, cyanotoxin-associated BGC screening, natural competence and R–M profiling and B_12_-related genomic and uptake analyses, the study defines a workflow for moving from strain identity to safety assessment, genetic tractability and functional validation. The defined workflow is increasingly important for cyanobacteria and other micro-organisms being considered for biotechnology, where strain identity, safety and functional performance must be assessed together. The findings of the present study strengthen the foundation for selecting, characterizing and engineering *AFA* and related *Nostocales* for nutritional biotechnology, sustainable biomass production and future cyanobacterial synthetic biology.

## Supplementary material

10.1099/mgen.0.001832 Supplementary Material 1.

10.1099/mgen.0.001832 Supplementary Material 2.

## Abbreviations

ADA: *Aphanizomenon*, *Dolichospermum* and *Anabaena*
AFA: *Aphanizomenon flos-aquae*
ANI: average nucleotide identity
ATX: anatoxin
BGC: biosynthetic gene cluster
CYR: cylindrospermopsin
DMB: 5,6-dimethylbenzimidazole
GTDB-Tk: Genome Taxonomy Database Toolkit
HAB: harmful algal bloom
JW: Jaworski medium
KEGG: Kyoto Encyclopedia of Genes and Genomes
LC-MS: Liquid chromatography-mass spectrometry
MAG: metagenome-assembled genome
MYC: microcystin
NCBI: National Center for Biotechnology Information
NOD: nodularin
REN: restriction endonuclease
R–M: restriction–modification
SXT: saxitoxin
WGS: whole-genome sequencing

## References

[1] Veaudor T, Blanc-Garin V, Chenebault C, Diaz-Santos E, Sassi J-F (2020). Recent advances in the photoautotrophic metabolism of cyanobacteria: biotechnological implications. Life (Basel).

[2] Cirés S, Ballot A (2016). A review of the phylogeny, ecology and toxin production of bloom-forming *Aphanizomenon* spp. and related species within the *Nostocales* (*Cyanobacteria*). Harmful Algae.

[3] Heimann K, Cirés S (2015). Handbook of Marine Microalgae.

[4] Garg R, Maldener I (2021). The formation of spore-like akinetes: a survival strategy of filamentous cyanobacteria. *Microb Physiol*.

[5] Kumar J, Singh D, Tyagi MB, Kumar A (2019). Cyanobacteria.

[6] Carmichael WW, Drapeau C, Anderson DM (2000). Harvesting of *Aphanizomenon flos-aquae* Ralfs ex Born. J Appl Phycol.

[7] Benedetti S, Benvenuti F, Pagliarani S, Francogli S, Scoglio S (2004). Antioxidant properties of a novel phycocyanin extract from the blue-green alga *Aphanizomenon flos-aquae*. Life Sci.

[8] Nuzzo D, Presti G, Picone P, Galizzi G, Gulotta E (2018). Effects of the *Aphanizomenon flos-aquae* extract (Klamin®) on a neurodegeneration cellular model. Oxid Med Cell Longev.

[9] Scoglio GD, Jackson HO, Purton S (2024). The commercial potential of *Aphanizomenon flos-aquae*, a nitrogen-fixing edible cyanobacterium. J Appl Phycol.

[10] Miller TR, Xiong A, Deeds JR, Stutts WL, Samdal IA (2020). Microcystin toxins at potentially hazardous levels in algal dietary supplements revealed by a combination of bioassay, immunoassay, and mass spectrometric methods. J Agric Food Chem.

[11] Scoglio S, Scoglio GD (2024). Klamath lake *Aphanizomenon flos-aquae*: wild-harvesting, extracts and benefits. IntechOpen.

[12] Dreher TW, Davis EW, Mueller RS (2021). Complete genomes derived by directly sequencing freshwater bloom populations emphasize the significance of the genus level ADA clade within the *Nostocales*. Harmful Algae.

[13] Nowruzi B, Porzani SJ (2021). Toxic compounds produced by cyanobacteria belonging to several species of the order Nostocales: a review. J Appl Toxicol.

[14] Österholm J, Popin RV, Fewer DP, Sivonen K (2020). Phylogenomic analysis of secondary metabolism in the toxic cyanobacterial genera *Anabaena, Dolichospermum* and *Aphanizomenon*. *Toxins*.

[15] Li R, Carmichael WW, Liu Y, Watanabe MM (2000). Taxonomic re-evaluation of *Aphanizomenon flos-aquae* NH-5 based on morphology and 16S rRNA gene sequences. Hydrobiologia.

[16] Komárek J (2010). Modern taxonomic revision of planktic nostocacean cyanobacteria: a short review of genera. Hydrobiologia.

[17] Rajaniemi P, Hrouzek P, Kaštovská K, Willame R, Rantala A (2005). Phylogenetic and morphological evaluation of the genera *Anabaena, Aphanizomenon, Trichormus* and *Nostoc* (Nostocales, Cyanobacteria). Int J Syst Evol Microbiol.

[18] Driscoll CB, Meyer KA, Šulčius S, Brown NM, Dick GJ (2018). A closely-related clade of globally distributed bloom-forming cyanobacteria within the *Nostocales*. Harmful Algae.

[19] Dreher TW, Davis EW, Mueller RS, Otten TG (2021). Comparative genomics of the ADA clade within the *Nostocales*. Harmful Algae.

[20] Wacklin P, Hoffmann L, Komárek J (2009). Nomenclatural validation of the genetically revised cyanobacterial genus *Dolichospermum* (Ralfs ex Bornet et Flahault) comb. nova. Fottea.

[21] Komárek J (2013). Süßwasserflora von Mitteleuropa, Bd. 19/3: Cyanoprokaryota 3.

[22] Bhat TA, Ahmad L, Ganai MA, Khan O (2015). Nitrogen fixing biofertilizers; mechanism and growth promotion: a review. J Pure Appl Microbiol.

[23] Chaurasia AK, Apte SK (2011). Improved eco-friendly recombinant *Anabaena* sp. strain PCC7120 with enhanced nitrogen biofertilizer potential. Appl Environ Microbiol.

[24] Chittora D, Meena M, Barupal T, Swapnil P, Sharma K (2020). Cyanobacteria as a source of biofertilizers for sustainable agriculture. Biochem Biophys Rep.

[25] Al-Haj L, Lui YT, Abed RMM, Gomaa MA, Purton S (2016). Cyanobacteria as chassis for industrial biotechnology: progress and prospects. Life (Basel).

[26] Nies F, Springstein BL, Hanke DM, Dagan T (2022). Natural competence in the filamentous, heterocystous cyanobacterium *Chlorogloeopsis fritschii* PCC 6912. mSphere.

[27] Schirmacher AM, Hanamghar SS, Zedler JAZ (2020). Function and Benefits of natural competence in cyanobacteria: from ecology to targeted manipulation. Life.

[28] Lähteenmäki-Uutela A, Rahikainen M, Lonkila A, Yang B (2021). Alternative proteins and EU food law. Food Control.

[29] Schaeffer DJ, Malpas PB, Barton LL (1999). Risk assessment of microcystin in dietary *Aphanizomenon flos-aquae*. Ecotoxicol Environ Saf.

[30] Elhai J, Thiel T, Pakrasi HB (1990). DNA transfer into cyanobacteria. Plant Mol Biol Man.

[31] Gale GAR, Schiavon Osorio AA, Puzorjov A, Wang B, McCormick AJ (2019). Genetic modification of cyanobacteria by conjugation using the cyanogate modular cloning toolkit. J Vis Exp.

[32] Helliwell KE, Lawrence AD, Holzer A, Kudahl UJ, Sasso S (2016). Cyanobacteria and eukaryotic algae use different chemical variants of vitamin B12. Curr Biol.

[33] Miyamoto E, Tanioka Y, Nakao T, Barla F, Inui H (2006). Purification and characterization of a corrinoid-compound in an edible cyanobacterium *Aphanizomenon flos-aquae* as a nutritional supplementary food. J Agric Food Chem.

[34] Balabanova L, Averianova L, Marchenok M, Son O, Tekutyeva L (2021). Microbial and genetic resources for cobalamin (Vitamin B12) biosynthesis: from ecosystems to industrial biotechnology. Int J Mol Sci.

[35] Pérez AA, Rodionov DA, Bryant DA (2016). Identification and regulation of genes for cobalamin transport in the cyanobacterium *Synechococcus* sp. Strain PCC 7002. J Bacteriol.

[36] Jaworski GHM, Talling JF, Heaney SI (1981). The influence of carbon dioxide-depletion on growth and sinking rate of two planktonic diatoms in culture. British Phycological J.

[37] Saker ML, Jungblut A-D, Neilan BA, Rawn DFK, Vasconcelos VM (2005). Detection of microcystin synthetase genes in health food supplements containing the freshwater cyanobacterium *Aphanizomenon flos-aquae*. Toxicon.

[38] Uritskiy GV, DiRuggiero J, Taylor J (2018). MetaWRAP-a flexible pipeline for genome-resolved metagenomic data analysis. Microbiome.

[39] Bankevich A, Nurk S, Antipov D, Gurevich AA, Dvorkin M (2012). SPAdes: a new genome assembly algorithm and its applications to single-cell sequencing. J Comput Biol.

[40] Alneberg J, Bjarnason BS, de Bruijn I, Schirmer M, Quick J (2014). Binning metagenomic contigs by coverage and composition. Nat Methods.

[41] Wu Y-W, Simmons BA, Singer SW (2016). MaxBin 2.0: an automated binning algorithm to recover genomes from multiple metagenomic datasets. Bioinformatics.

[42] Kang DD, Li F, Kirton E, Thomas A, Egan R (2019). MetaBAT 2: an adaptive binning algorithm for robust and efficient genome reconstruction from metagenome assemblies. PeerJ.

[43] Parks DH, Imelfort M, Skennerton CT, Hugenholtz P, Tyson GW (2015). CheckM: assessing the quality of microbial genomes recovered from isolates, single cells, and metagenomes. Genome Res.

[44] Kolmogorov M, Bickhart DM, Behsaz B, Gurevich A, Rayko M (2020). metaFlye: scalable long-read metagenome assembly using repeat graphs. Nat Methods.

[45] Liu C-C, Dong S-S, Chen J-B, Wang C, Ning P (2022). MetaDecoder: a novel method for clustering metagenomic contigs. Microbiome.

[46] Walker BJ, Abeel T, Shea T, Priest M, Abouelliel A (2014). Pilon: an integrated tool for comprehensive microbial variant detection and genome assembly improvement. PLoS One.

[47] Langmead B, Salzberg SL (2012). Fast gapped-read alignment with Bowtie 2. Nat Methods.

[48] Seemann T (2014). Prokka: rapid prokaryotic genome annotation. Bioinformatics.

[49] Carver T, Harris SR, Berriman M, Parkhill J, McQuillan JA (2012). Artemis: an integrated platform for visualization and analysis of high-throughput sequence-based experimental data. Bioinformatics.

[50] Ruiz-Perez CA, Conrad RE, Konstantinidis KT (2021). MicrobeAnnotator: a user-friendly, comprehensive functional annotation pipeline for microbial genomes. BMC Bioinformatics.

[51] Virtanen P, Gommers R, Oliphant TE, Haberland M, Reddy T (2020). SciPy 1.0: fundamental algorithms for scientific computing in Python. Nat Methods.

[52] Cantalapiedra CP, Hernández-Plaza A, Letunic I, Bork P, Huerta-Cepas J (2021). eggNOG-mapper v2: functional annotation, orthology assignments, and domain prediction at the metagenomic scale. Mol Biol Evol.

[53] Bateman A, Birney E, Cerruti L, Durbin R, Etwiller L (2002). The Pfam protein families database. Nucleic Acids Res.

[54] Kanehisa M, Furumichi M, Tanabe M, Sato Y, Morishima K (2017). KEGG: new perspectives on genomes, pathways, diseases and drugs. Nucleic Acids Res.

[55] Blin K, Shaw S, Augustijn HE, Reitz ZL, Biermann F (2023). antiSMASH 7.0: new and improved predictions for detection, regulation, chemical structures and visualisation. Nucleic Acids Res.

[56] Segata N, Börnigen D, Morgan XC, Huttenhower C (2013). PhyloPhlAn is a new method for improved phylogenetic and taxonomic placement of microbes. Nat Commun.

[57] Stamatakis A (2014). RAxML version 8: a tool for phylogenetic analysis and post-analysis of large phylogenies. Bioinformatics.

[58] Chaumeil P-A, Mussig AJ, Hugenholtz P, Parks DH (2022). GTDB-Tk v2: memory friendly classification with the genome taxonomy database. Bioinformatics.

[59] Jain C, Rodriguez-R LM, Phillippy AM, Konstantinidis KT, Aluru S (2018). High throughput ANI analysis of 90K prokaryotic genomes reveals clear species boundaries. Nat Commun.

[60] Altschul SF, Madden TL, Schäffer AA, Zhang J, Zhang Z (1997). Gapped BLAST and PSI-BLAST: a new generation of protein database search programs. Nucleic Acids Res.

[61] Nawrocki EP, Eddy SR (2013). Infernal 1.1: 100-fold faster RNA homology searches. Bioinformatics.

[62] Kalvari I, Nawrocki EP, Argasinska J, Quinones-Olvera N, Finn RD (2018). Non-Coding RNA analysis using the rfam database. Curr Protoc Bioinformatics.

[63] Schwengers O, Jelonek L, Dieckmann MA, Beyvers S, Blom J (2021). Bakta: rapid and standardized annotation of bacterial genomes via alignment-free sequence identification. Microb Genom.

[64] Bunbury F, Deery E, Sayer AP, Bhardwaj V, Harrison EL (2022). Exploring the onset of b(12) -based mutualisms using a recently evolved chlamydomonas auxotroph and b(12) -producing bacteria. Environ Microbiol.

[65] Lawrence AD, Nemoto-Smith E, Deery E, Baker JA, Schroeder S (2018). Construction of fluorescent analogs to follow the uptake and distribution of cobalamin (Vitamin B_12_) in Bacteria, Worms, and Plants. Cell Chem Biol.

[66] Laamanen MJ, Forsström L, Sivonen K (2002). Diversity of *Aphanizomenon flos-aquae* (cyanobacterium) populations along a Baltic Sea salinity gradient. Appl Environ Microbiol.

[67] Dolman AM, Rücker J, Pick FR, Fastner J, Rohrlack T (2012). Cyanobacteria and cyanotoxins: the influence of nitrogen versus phosphorus. PLoS One.

[68] Cusick KD, Sayler GS (2013). An overview on the marine neurotoxin, saxitoxin: genetics, molecular targets, methods of detection and ecological functions. Mar Drugs.

[69] Wiese M, D’Agostino PM, Mihali TK, Moffitt MC, Neilan BA (2010). Neurotoxic alkaloids: saxitoxin and its analogs. Mar Drugs.

[70] Zurawell RW, Chen H, Burke JM, Prepas EE (2005). Hepatotoxic cyanobacteria: a review of the biological importance of microcystins in freshwater environments. J Toxicol Environ Health B.

[71] Bashir F, Bashir A, Bouaïcha N, Chen L, Codd GA (2023). Cyanotoxins, biosynthetic gene clusters, and factors modulating cyanotoxin biosynthesis. World J Microbiol Biotechnol.

[72] Brown NM, Mueller RS, Shepardson JW, Landry ZC, Morré JT (2016). Structural and functional analysis of the finished genome of the recently isolated toxic *Anabaena* sp. WA102. *BMC Genomics*.

[73] Kust A, Méjean A, Ploux O (2020). Biosynthesis of anatoxins in *Cyanobacteria*: identification of the carboxy-anatoxins as the penultimate biosynthetic intermediates. J Nat Prod.

[74] Neilan BA, Pearson LA, Muenchhoff J, Moffitt MC, Dittmann E (2013). Environmental conditions that influence toxin biosynthesis in cyanobacteria. Environ Microbiol.

[75] Rantala-Ylinen A, Känä S, Wang H, Rouhiainen L, Wahlsten M (2011). Anatoxin-a synthetase gene cluster of the cyanobacterium Anabaena sp. strain 37 and molecular methods to detect potential producers. Appl Environ Microbiol.

[76] Méjean A, Mann S, Maldiney T, Vassiliadis G, Lequin O (2009). Evidence that biosynthesis of the neurotoxic alkaloids anatoxin-a and homoanatoxin-a in the cyanobacterium *Oscillatoria* PCC 6506 occurs on a modular polyketide synthase initiated by L-proline. J Am Chem Soc.

[77] Mihali TK, Carmichael WW, Neilan BA (2011). A putative gene cluster from a *Lyngbya wollei* bloom that encodes paralytic shellfish toxin biosynthesis. PLoS One.

[78] Mahmood NA, Carmichael WW (1986). Paralytic shellfish poisons produced by the freshwater cyanobacterium *Aphanizomenon flos*-aquae NH-5. Toxicon.

[79] Kellmann R, Mihali TK, Jeon YJ, Pickford R, Pomati F (2008). Biosynthetic intermediate analysis and functional homology reveal a saxitoxin gene cluster in cyanobacteria. Appl Environ Microbiol.

[80] McCall JR, Holland WC, Keeler DM, Hardison DR, Litaker RW (2019). Improved accuracy of saxitoxin measurement using an optimized enzyme-linked immunosorbent assay. *Toxins (Basel*).

[81] Moffitt MC, Neilan BA (2004). Characterization of the nodularin synthetase gene cluster and proposed theory of the evolution of cyanobacterial hepatotoxins. Appl Environ Microbiol.

[82] Fewer DP, Jokela J, Rouhiainen L, Wahlsten M, Koskenniemi K (2009). The non-ribosomal assembly and frequent occurrence of the protease inhibitors spumigins in the bloom-forming cyanobacterium *Nodularia spumigena*. Mol Microbiol.

[83] Jokela J, Heinilä LMP, Shishido TK, Wahlsten M, Fewer DP (2017). Production of high amounts of hepatotoxin nodularin and new protease inhibitors pseudospumigins by the Brazilian benthic *Nostoc* sp. CENA543. Front Microbiol.

[84] Pearson L, Mihali T, Moffitt M, Kellmann R, Neilan B (2010). On the chemistry, toxicology and genetics of the cyanobacterial toxins, microcystin, nodularin, saxitoxin and cylindrospermopsin. Mar Drugs.

[85] Zhou C, Chen H, Zhao H, Wang Q (2021). Microcystin biosynthesis and toxic effects. Algal Research.

[86] Kaloudis T, Zervou S-K, Tsimeli K, Triantis TM, Fotiou T (2013). Determination of microcystins and nodularin (cyanobacterial toxins) in water by LC-MS/MS. Monitoring of Lake Marathonas, a water reservoir of Athens, Greece. J Hazard Mater.

[87] Chorus I, Fastner J, Welker M (2021). Cyanobacteria and cyanotoxins in a changing environment: concepts, controversies, challenges. Water.

[88] McGregor GB, Sendall BC (2017). *Iningainema pulvinus* gen nov., sp nov. (Cyanobacteria, Scytonemataceae) a new nodularin producer from Edgbaston Reserve, north-eastern Australia. Harmful Algae.

[89] Scarlett KR, Kim S, Lovin LM, Chatterjee S, Scott JT (2020). Global scanning of cylindrospermopsin: critical review and analysis of aquatic occurrence, bioaccumulation, toxicity and health hazards. Sci Total Environ.

[90] Wendt KE, Pakrasi HB (2019). Genomics approaches to deciphering natural transformation in cyanobacteria. Front Microbiol.

[91] Jester BW, Zhao H, Gewe M, Adame T, Perruzza L (2022). Development of spirulina for the manufacture and oral delivery of protein therapeutics. Nat Biotechnol.

[92] Mell JC, Redfield RJ (2014). Natural competence and the evolution of DNA uptake specificity. J Bacteriol.

[93] Blokesch M (2016). Natural competence for transformation. Curr Biol.

[94] Nies F, Mielke M, Pochert J, Lamparter T (2020). Natural transformation of the filamentous cyanobacterium *Phormidium lacuna*. PLoS One.

[95] Heidorn T, Camsund D, Huang H-H, Lindberg P, Oliveira P (2011). Methods in Enzymology.

[96] Stucken K, Koch R, Dagan T (2013). Cyanobacterial defense mechanisms against foreign DNA transfer and their impact on genetic engineering. Biol Res.

[97] Clerico EM, Ditty JL, Golden SS (2007). Specialized techniques for site-directed mutagenesis in cyanobacteria. Methods Mol Biol.

[98] Wolk CP, Vonshak A, Kehoe P, Elhai J (1984). Construction of shuttle vectors capable of conjugative transfer from *Escherichia coli* to nitrogen-fixing filamentous cyanobacteria. Proc Natl Acad Sci USA.

[99] Elhai J, Wolk CP (1988). Conjugal transfer of DNA to cyanobacteria. *Methods Enzymol*.

[100] Koksharova OA, Wolk CP (2002). Genetic tools for cyanobacteria. Appl Microbiol Biotechnol.

[101] Wang H, Sivonen K, Rouhiainen L, Fewer DP, Lyra C (2012). Genome-derived insights into the biology of the hepatotoxic bloom-forming cyanobacterium *Anabaena* sp. strain 90. BMC Genomics.

[102] Shaw LP, Rocha EPC, MacLean RC (2023). Restriction-modification systems have shaped the evolution and distribution of plasmids across bacteria. Nucleic Acids Res.

[103] Curtain F, Grafenauer S (2019). Plant-based meat substitutes in the flexitarian age: an audit of products on supermarket shelves. Nutrients.

[104] Watanabe F, Bito T (2018). Vitamin b(12) sources and microbial interaction. Exp Biol Med.

[105] Taga ME, Walker GC (2008). Pseudo-B12 joins the cofactor family. J Bacteriol.

[106] Lechuga Morente D (2021). A first study for the establishment of a source of vitamin B12 as of the genetic modification of the cyanobacteria arthrospira platensis.

[107] Pérez AA, Liu Z, Rodionov DA, Li Z, Bryant DA (2016). Complementation of cobalamin auxotrophy in *Synechococcus* sp. strain PCC 7002 and validation of a putative cobalamin riboswitch *In Vivo*. J Bacteriol.

[108] Goudsmits JMH, Slotboom DJ, van Oijen AM (2017). Single-molecule visualization of conformational changes and substrate transport in the vitamin B_12_ ABC importer BtuCD-F. Nat Commun.

[109] Cadieux N, Bradbeer C, Reeger-Schneider E, Köster W, Mohanty AK (2002). Identification of the periplasmic cobalamin-binding protein BtuF of *Escherichia coli*. J Bacteriol.

[110] Lewinson O, Lee AT, Locher KP, Rees DC (2010). A distinct mechanism for the ABC transporter BtuCD–BtuF revealed by the dynamics of complex formation. *Nat Struct Mol Biol*.

[111] Fowler CC, Brown ED, Li Y (2010). Using a riboswitch sensor to examine coenzyme B12 metabolism and transport in *E. coli*. Chemistry & Biology.

[112] Croft MT, Lawrence AD, Raux-Deery E, Warren MJ, Smith AG (2005). Algae acquire vitamin B12 through a symbiotic relationship with bacteria. Nature.

[113] Driscoll CB (2016). Comparative Genomics of Freshwater Bloom-Forming Cyanobacteria and Associated Organisms.

[114] Driscoll CB, Otten TG, Brown NM, Dreher TW (2017). Towards long-read metagenomics: complete assembly of three novel genomes from bacteria dependent on a diazotrophic cyanobacterium in a freshwater lake co-culture. *Stand in Genomic Sci*.

[115] Morris JJ, Lenski RE, Zinser ER (2012). The black queen hypothesis: evolution of dependencies through adaptive gene loss. mBio.

[116] Baroni L, Scoglio S, Benedetti S, Bonetto C, Pagliarani S (2009). Effect of a Klamath algae product (“AFA-B12”) on blood levels of vitamin B12 and homocysteine in vegan subjects: a pilot study. Int J Vitam Nutr Res.

[117] Stüken A, Jakobsen KS (2010). The cylindrospermopsin gene cluster of *Aphanizomenon* sp. strain 10E6: organization and recombination. Microbiology.

[118] Tillett D, Dittmann E, Erhard M, von Döhren H, Börner T (2000). Structural organization of microcystin biosynthesis in *Microcystis aeruginosa* PCC7806: an integrated peptide–polyketide synthetase system. Chem Biol.

