# Supplementary material for: Genomic insights into the taxonomy, safety and biotechnological potential of Aphanizomenon flos-aquae and related Nostocales
Source: Microb Genom. 2026 Sep 23;12(9):001832. doi: 10.1099/mgen.0.001832 (PMC13603067; doi:10.1099/mgen.0.001832)
Supplement: Supplementary file 1 [file mgen-12-01832-s001.pdf]

Genomic insights into the taxonomy, safety and biotechnological potential of *Aphanizomenon flos-aquae* and related Nostocales

Gabriel D. Scoglio, David H. Green, Harry O. Jackson, and Saul Purton

List of supplementary files

Supplementary File 1: Comprehensive merged annotation file for the 164 *Nostocaceae* genomes analysed in this study. The file contains all CDS annotations generated from eggNOG, Pfam, KEGG, dcan, and antiSMASH. Owing to its size, opening the file in Excel is possible but not recommended; parsing the file in R or via Unix command line for efficient keyword searches and downstream analyses is recommended.

Download link:
https://drive.google.com/file/d/1zZy-Q2XnJ62LAh_YSflEkzMjcq8ziwjS/view?usp=sharing

Supplementary Figure 1

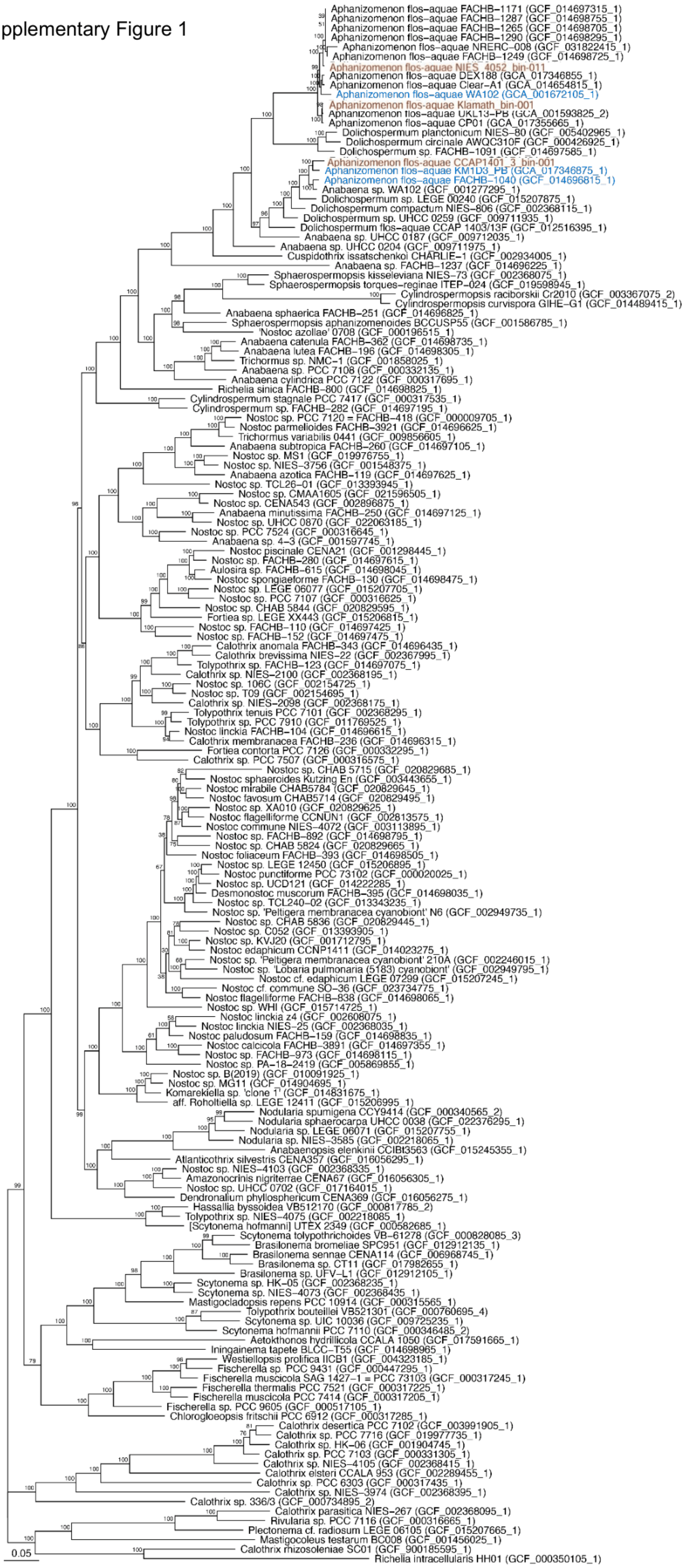

Supplementary Figure 1: Maximum-likelihood phylogenetic tree of the 164 analysed genomes reconstructed with PhyloPhlAn v3.0.60 and RAxML v8.2.12. Organism labels follow the NCBI/traditional names used throughout the manuscript for consistency with accession records and the cyanobacterial literature. GTDB-Tk classifications were generated for all genomes and are provided separately in Suppl. Table 13 as genome-based taxonomic support. Newly sequenced *AFA* genomes are highlighted in red, while *AFA*-labelled genomes requiring taxonomic reassessment are shown in blue. Bootstrap support values are indicated at the nodes. The scale bar represents substitutions per site.

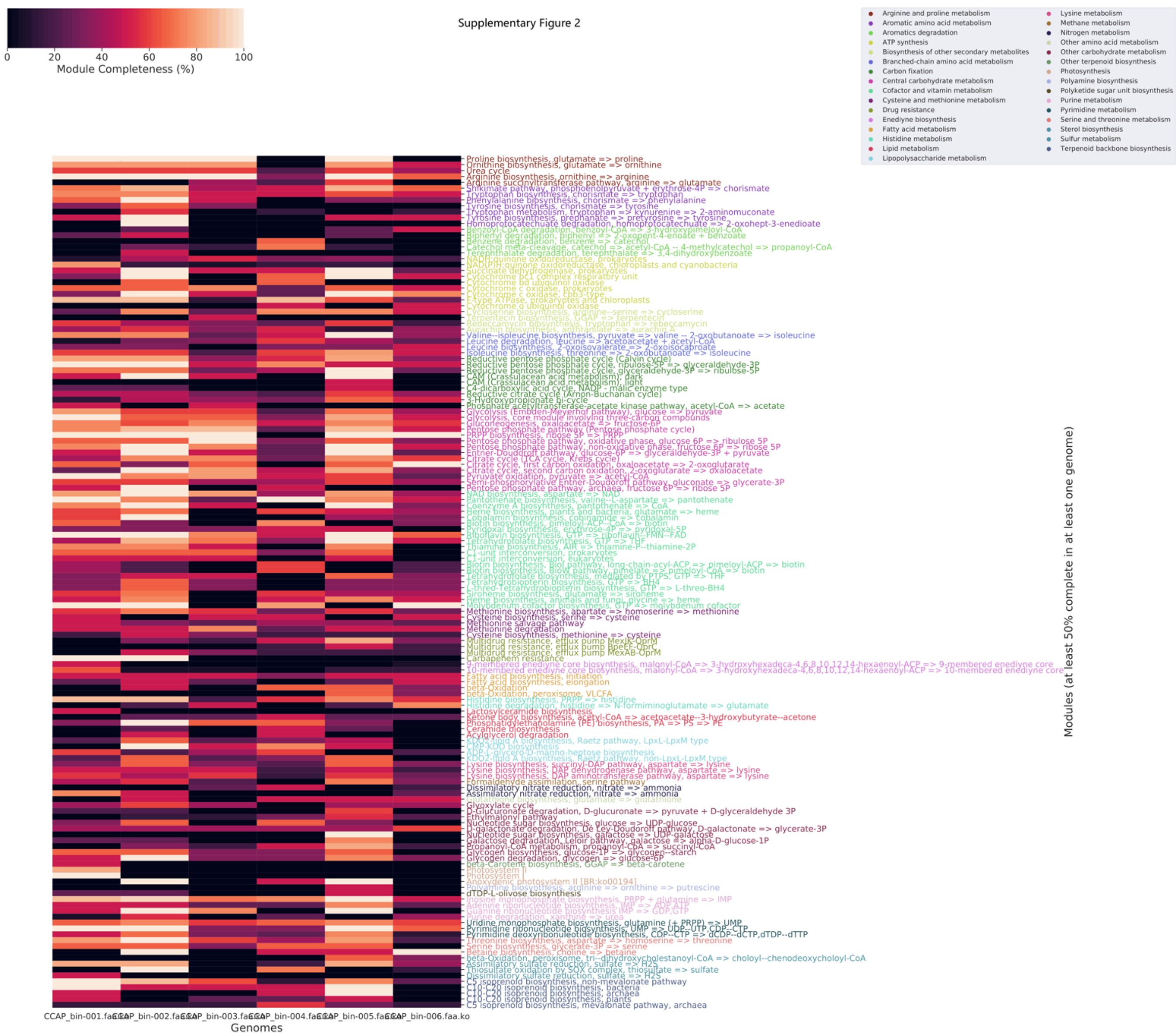

Supplementary Figure 2: Metabolic reconstruction and comparison of CCAP 1401/3 *AFA* and its associated metagenome-assembled bacterial genomes. Functional annotation was performed using KEGG modules and summarised as heatmaps, where colours, from black (0%) to white (100%), indicate module completeness – i.e. the percentage of genes present that are required for each pathway. The panels depict representative pathways across major functional categories, such amino acid biosynthesis and degradation, central carbon and nitrogen metabolism, energy production; cofactor and vitamin-associated pathways.

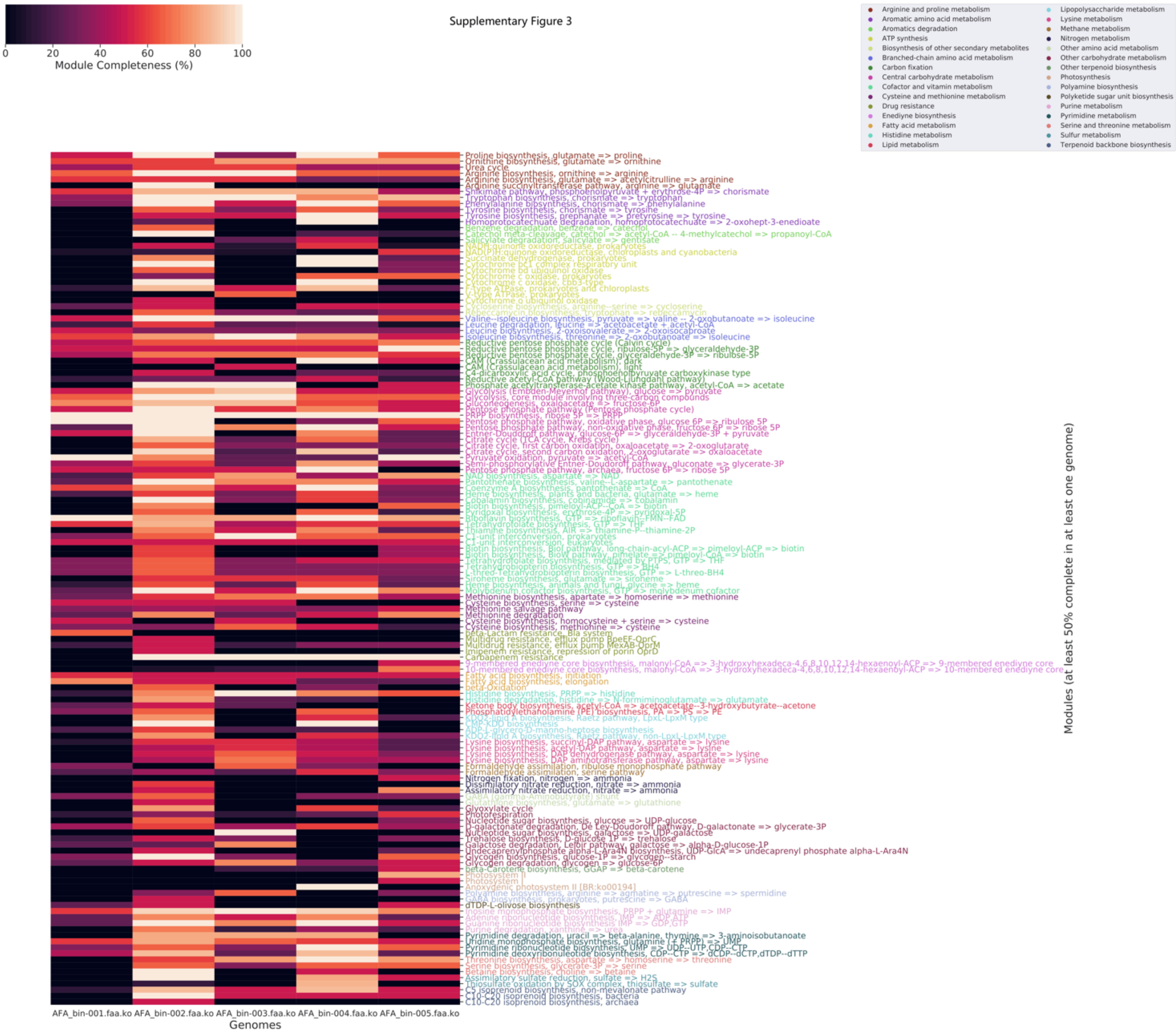

Supplementary Figure 3: Metabolic reconstruction and comparison of Klamath *AFA* and its associated metagenome-assembled bacterial genomes. Functional annotation was performed using KEGG modules and summarised as heatmaps, where colours, from black (0%) to white (100%), indicate module completeness – i.e. the percentage of genes present that are required for each pathway. The panels depict representative pathways across major functional categories, such amino acid biosynthesis and degradation, central carbon and nitrogen metabolism, energy production; cofactor and vitamin-associated pathways.

Supplementary Figure 4

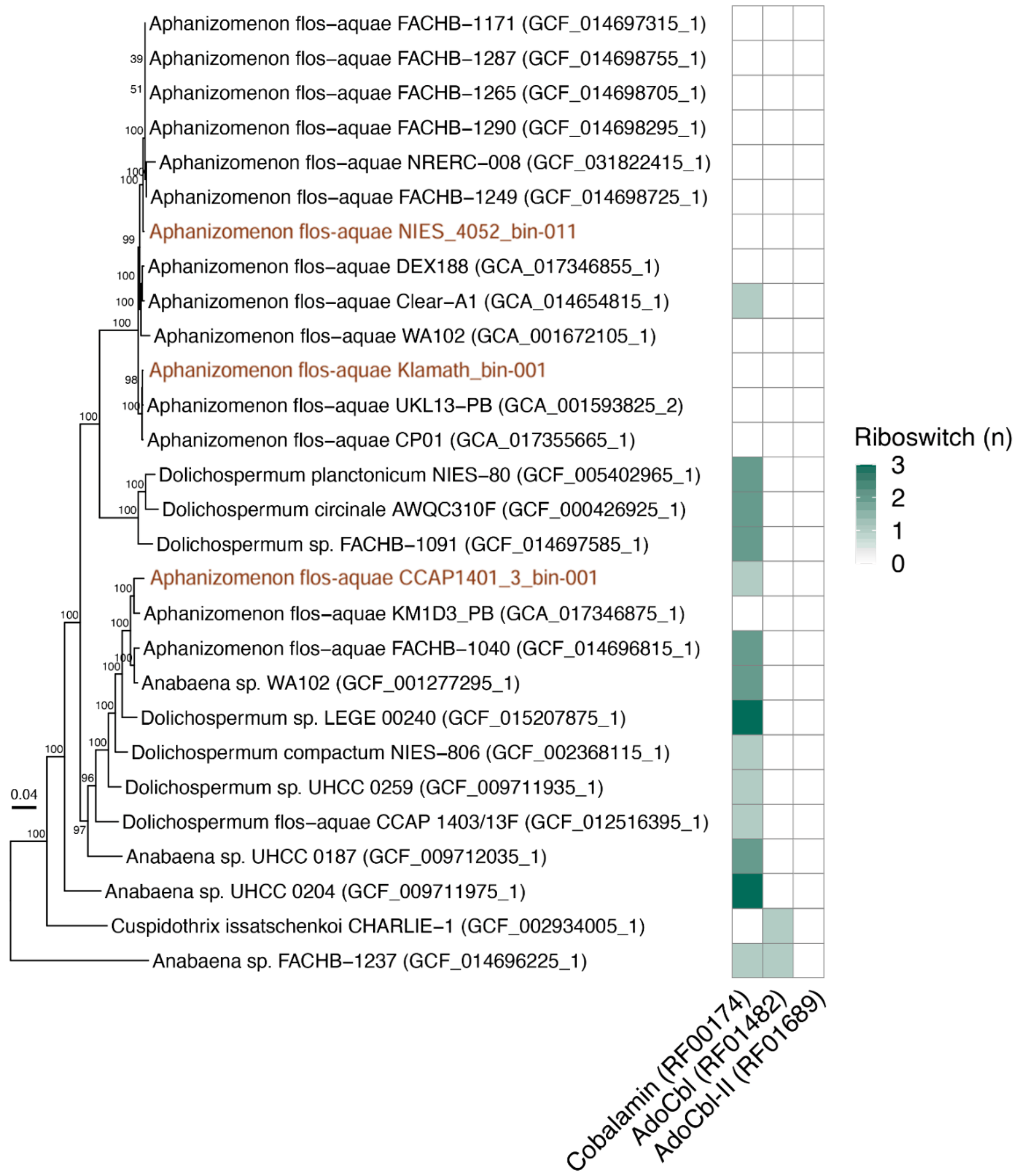

Supplementary Figure 4: Distribution of predicted cobalamin-related riboswitches across the *AFA/ADA* clade. The maximum-likelihood phylogenetic tree shows *AFA*-labelled genomes and closely related *Aphanizomenon*, *Dolichospermum*, *Anabaena* and *Cuspidothrix* genomes. Newly sequenced AFA genomes are highlighted in red. The adjacent heatmap shows the number of predicted cobalamin-related RNA regulatory elements detected per genome using Infernal/Rfam searches. Columns correspond to cobalamin riboswitches (RF00174), AdoCbl riboswitches (RF01482) and AdoCbl variant riboswitches (RF01689). Colour intensity indicates riboswitch count per genome. Bootstrap support values are indicated at the nodes where displayed, and the scale bar represents substitutions per site.
